# NsrR1, a Nitrogen Stress-Repressed sRNA, Contributes to the Regulation of *nblA* in *Nostoc* sp. PCC 7120

**DOI:** 10.3389/fmicb.2018.02267

**Published:** 2018-09-24

**Authors:** Isidro Álvarez-Escribano, Agustín Vioque, Alicia M. Muro-Pastor

**Affiliations:** Instituto de Bioquímica Vegetal y Fotosíntesis, Consejo Superior de Investigaciones Científicas and Universidad de Sevilla, Seville, Spain

**Keywords:** regulatory RNA, cyanobacteria, NtcA, feed-forward loop, post-transcriptional regulation

## Abstract

Small regulatory RNAs (sRNAs) are currently considered as major post-transcriptional regulators of gene expression in bacteria. The interplay between sRNAs and transcription factors leads to complex regulatory networks in which both transcription factors and sRNAs may appear as nodes. In cyanobacteria, the responses to nitrogen availability are controlled at the transcriptional level by NtcA, a CRP/FNR family regulator. In this study, we describe an NtcA-regulated sRNA in the cyanobacterium *Nostoc* sp. PCC 7120, that we have named NsrR1 (nitrogen stress repressed RNA1). We show sequence specific binding of NtcA to the promoter of NsrR1. Prediction of possible mRNA targets regulated by NsrR1 allowed the identification of *nblA*, encoding a protein adaptor for phycobilisome degradation under several stress conditions, including nitrogen deficiency. We demonstrate specific interaction between NsrR1 and the 5′-UTR of the *nblA* mRNA, that leads to decreased expression of *nblA*. Because both NsrR1 and NblA are under transcriptional control of NtcA, this regulatory circuit constitutes a coherent feed-forward loop, involving a transcription factor and an sRNA.

## Introduction

Small non-coding RNAs (sRNAs) are important players in bacterial regulatory networks. Most often, this type of molecules exert its function at the post-transcriptional level by fine-tuning responses to different environmental situations (Wagner and Romby, 2015). The interplay between transcription factors and small RNAs provides an additional level of control that, in many cases, results in feed-forward regulatory loops (coherent or incoherent) between a transcription factor, an sRNA and the regulated target (Mandin and Guillier, 2013; Nitzan et al., 2017).

Global nitrogen regulation in cyanobacteria is controlled by NtcA, a protein that belongs to the CRP/FNR family of transcriptional regulators (Herrero et al., 2001). Direct transcriptional regulation mediated by NtcA is operated by binding to conserved sequences (first defined as GTAN_8_TAC, later re-defined as GTN_10_AC) in the promoters of the regulated genes (Luque et al., 1994; Mitschke et al., 2011; Picossi et al., 2014; Giner-Lamia et al., 2017). NtcA has been described to act either as an activator or as a repressor of transcription, depending on the location of the binding site with respect to the regulated transcriptional start site (TSS) (Mitschke et al., 2011; Picossi et al., 2014). Most NtcA-activated promoters contain an NtcA binding site centered around position -41.5 with respect to the TSS (like Class II CRP-activated promoters). In contrast, NtcA-repressed genes bear NtcA binding sites that overlap critical elements of the promoter, including the -35 box, the -10 box or the TSS. Therefore, the mechanism of repression by NtcA involves interference with RNA polymerase binding. Similar to the case of other CRP-family regulators, NtcA has been shown to regulate expression of several sRNAs (Mitschke et al., 2011; Brenes-Álvarez et al., 2016) including NsiR4 (nitrogen-stress inducible RNA 4), which is involved in nitrogen assimilation control via regulation of the key enzyme glutamine synthetase (Klähn et al., 2015).

Non-diazotrophic cyanobacteria respond to nutrient deprivation by a process called chlorosis, involving degradation of photosynthetic pigments, including the phycobilisomes (PBS) (Grossman et al., 1993a,b). Degradation of PBS protects the photosynthetic apparatus under exposure to high light and also provides amino acids as a source of nitrogen under nitrogen deficiency. NblA (non-bleaching phenotype) is a protein adaptor involved in PBS degradation by targeting phycobiliproteins to a proteolytic complex (Karradt et al., 2008; Sendersky et al., 2014). The *nblA* gene is induced under stress situations that promote PBS degradation (Collier and Grossman, 1994). *nblA* has multiple promoters and is subject to complex regulation that includes transcription induction by NtcA under nitrogen deprivation (Luque et al., 2001; Mitschke et al., 2011) and regulation by FurA under iron deficiency (González et al., 2016). Although heterocystous cyanobacteria are ultimately able to overcome nitrogen deprivation by fixation of atmospheric nitrogen, their transient response to nitrogen deprivation also includes induction of the *nblA* gene and partial degradation of PBS (Baier et al., 2004; Karradt et al., 2008).

We have previously carried out a differential RNA-Seq-based genome-wide identification of TSSs in the heterocystous cyanobacteria *Nostoc* sp. PCC 7120 (also known as *Anabaena* sp. PCC 7120), which included a description of transcriptional responses corresponding to the NtcA regulon (Mitschke et al., 2011). Based on the RNA-Seq data, we have also conducted a global approach to the identification of conserved, potentially regulatory small non-coding RNAs (sRNAs) in *Nostoc* sp. PCC 7120. This work has lead to the prediction and verification of several phylogenetically conserved sRNAs, including nitrogen-regulated and heterocyst-specific sRNAs (Brenes-Álvarez et al., 2016).

In this work, we show transcription of a small, nitrogen-regulated RNA, which we have named NsrR1 (nitrogen stress-repressed RNA 1), in *Nostoc* sp. PCC 7120. The promoter of NsrR1 contains a putative NtcA binding site, whose position, overlapping the -10 box, is suggestive of NtcA-mediated repression. We demonstrate sequence specific binding of purified NtcA to the promoter of *nsrR1*. Phylogenetic analysis reveals that sequences encoding orthologs of NsrR1 and the nearby NtcA binding site overlapping the -10 box are conserved among heterocystous cyanobacteria. Computational target prediction for NsrR1 identified *nblA* mRNA as possibly interacting with NsrR1 and therefore being subjected to post-transcriptional regulation by this sRNA. Here, we verify that NsrR1 regulates expression of NblA by its interaction with the 5′-UTR of *nblA* mRNA, therefore participating in an NtcA-operated coherent feed-forward regulatory loop.

## Materials and Methods

### Strains and Growth Conditions

Cultures of wild-type and the different mutant strains of *Nostoc* sp. PCC 7120 (**Supplementary Table S1**) were bubbled with an air/CO_2_ mixture (1% v/v) and grown photoautotrophically at 30°C in BG11 medium (Rippka et al., 1979) containing ferric citrate instead of ammonium ferric citrate, lacking NaNO_3_ and containing 6 mM NH_4_Cl, 10 mM NaHCO_3_, and 12 mM *N*-tris(hydroxymethyl)methyl-2-aminoethanesulfonic acid-NaOH buffer (pH 7.5). Nitrogen deficiency was induced by removal of combined nitrogen. Occasionally, 17.6 mM NaNO_3_ was used as nitrogen source. Solid media were solidified with 1% Difco Agar. Mutant strains were grown in the presence of appropriate antibiotics at the following concentrations: streptomycin (Sm) and spectinomycin (Sp), 2–3 μg/ml each (liquid medium) or 5 μg/ml each (solid medium), neomycin (Nm), 5 μg/ml (liquid medium) or 25 μg/ml (solid medium).

*Escherichia coli* strains (**Supplementary Table S1**) were grown in LB medium, supplemented with appropriate antibiotics (Sambrook and Russell, 2001).

### Generation of NsrR1 Mutant Strains

To generate a strain lacking NsrR1 (Δ*nsrR1*), two overlapping fragments encompassing flanking sequences around NsrR1 were amplified by PCR using as template genomic DNA with oligonucleotides 170 and 171 and oligonucleotides 172 and 173, respectively (see **Supplementary Table S2** for oligonucleotide sequences and description). The resulting products were then used as templates for a third PCR with oligonucleotides 170 and 173 resulting in the fusion of both fragments and the deletion of the sequences encoding NsrR1. The fragment was cloned into pSpark (Canvax Biotech), rendering pSAM319 and its sequence was verified by sequencing (Eurofins Genomics) (see **Supplementary Table S3** for plasmids description). After digestion with BamHI at the sites provided by oligonucleotides 170 and 173, the fragment was cloned into BamHI-digested *sacB*-containing SmSp^R^ vector pCSRO (Merino-Puerto et al., 2010), rendering pSAM325, which was transferred to *Nostoc* sp. strain PCC 7120 by conjugation (Elhai and Wolk, 1988) with selection for resistance to Sm and Sp. Cultures of the exconjugants obtained were used to select for clones resistant to 5% sucrose (Cai and Wolk, 1990), and individual sucrose resistant colonies were checked by PCR. Clones lacking the deleted region were named Δ*nsrR1*.

To establish controlled expression of NsrR1 in *Nostoc*, the *nsrR1* gene was placed under control of the *petE* promoter, which mediates Cu^2+^-regulated transcription (Buikema and Haselkorn, 2001) and was cloned in a self-replicating plasmid. The *petE* promoter of *Nostoc* sp. PCC 7120 (genomic coordinates 278185 to 277848) was amplified with oligonucleotides 186 and 299 and fused to the *nsrR1* fragment amplified with oligonucleotides 184 and 185 by a third PCR using the two fragments as templates and oligonucleotides 184 and 299. The product was cloned into pSpark, rendering pSAM329 and its sequence was verified. After digestion with ClaI and XhoI at the sites provided by oligonucleotides 299 and 184, the fragment was cloned into pSAM221, rendering pIAE17. pIAE17 was introduced into the Δ*nsrR1* strain by conjugation as described above.

### Whole Cell Spectra

Wild type or Δ*nsrR1* cells were grown under standard conditions in the presence of NH_4_^+^ or subjected to nitrogen deficiency for the number of hours indicated in each case. Spectra of the different cultures where taken between 500 and 750 nm on a JASCO V-650 spectrophotometer and normalized for chlorophyll content by the absorbance at 680 nm after subtraction of absorbance at 750 nm. Absorbance at 635 nm and 680 nm were used to estimate phycocyanin and chlorophyll content, respectively.

### Computing Methods

The predicted sequences of NsrR1 and its putative homologs were obtained from Brenes-Álvarez et al. (2016) and multiple sequence alignment was generated with T-Coffee (Notredame et al., 2000) at the EMBL-EBI web server (Li et al., 2015).

Target prediction of NsrR1 was performed using CopraRNA (Wright et al., 2013, 2014) with the NsrR1 homologs of *Nostoc* sp. PCC 7120, *Anabaena cylindrica* PCC 7122, *Anabaena variabilis* ATCC 29413 (recently renamed *Trichormus variabilis* NIES-23), *Cylindrospermum stagnale* PCC 7417, *Nodularia spumigena* CCY9414, ‘*Nostoc azollae*’ 0708, *Nostoc punctiforme* PCC 73102, *Nostoc* sp. PCC 7524, *Rivularia* sp. PCC 7116, *Nostoc* sp. PCC 7107, and *Calothrix* sp. PCC 7507.

The interaction between NsrR1 and the *nblA* mRNA of diverse cyanobacteria was analyzed with IntaRNA (Mann et al., 2017).

### RNA Isolation and Northern Blot Analysis

RNA samples were isolated from cells collected at different times after removing combined nitrogen from the media. Total RNA was isolated using hot phenol as described (Mohamed and Jansson, 1989) with modifications (Brenes-Álvarez et al., 2016). Northern blot hybridization was performed as previously described (Muro-Pastor et al., 1999; Steglich et al., 2008). Strand specific ^32^P-labeled probes for Northern blot were prepared with Taq DNA polymerase using a PCR fragment (oligonucleotides 158 and 159) as template in a reaction with α-^32^P-dCTP and one single oligonucleotide as primer (corresponding to the complementary strand of the sRNA or mRNA to be detected). Hybridization signals were quantified on a Cyclone Storage Phosphor System with Optiquant software.

### Expression and Purification of NtcA and NblA Proteins, EMSA Assays, and Western Blot

To produce His-tagged NtcA protein, the *ntcA* gene was amplified using *Nostoc* DNA as template and primers 343 and 344, and the PCR product was cloned in vector pET-28a (+) (Novagen) using NcoI and XhoI, producing plasmid pSAM334 (see **Supplementary Table S3** for plasmids description). Plasmid pSAM334, containing the *ntcA* gene fused to a sequence encoding a His_6_-tag under a T7 polymerase-dependent promoter, was transferred by electroporation to *E. coli* BL21-(DE3)-RIL, in which the gene encoding T7 RNA polymerase is under the control of an isopropyl-β-D-1-thiogalactopyranoside (IPTG)-regulated promoter. A 25-ml pre-inoculum of this strain was grown overnight in LB medium supplemented with chloramphenicol and kanamycin and used to inoculate 275 ml of the same medium. The culture was incubated at 37°C until OD_600_ = 0.6. Recombinant NtcA expression was induced by addition of 1 mM IPTG. After 4 h at 37°C, cells were collected and resuspended in 20 mM sodium phosphate buffer (pH 7.2) containing 500 mM NaCl, 20 mM imidazole and 1 mM phenylmethylsulfonyl fluoride (6 ml/g of cells). Cells were broken by sonication and after centrifugation at 15,000 × *g* (15 min, 4°C), the His_6_-NtcA protein was purified from the supernatant by chromatography through a 1-ml HisTrap HP column (GE Healthcare), using imidazole to elute the retained proteins. Samples obtained after purification were subjected to SDS-PAGE to assess purity and 10% glycerol was added to fractions before storage at -20°C.

A similar procedure was followed for expression and purification of NblA using oligonucleotides 358 and 359 for cloning of the *nblA* gene in pET-28a (+), rendering pIAE32. In this case, an additional purification step was carried out by size-exclusion chromatography in a HiLoad 16/60 Superdex 75 column (Pharmacia) using 20 mM sodium phosphate buffer (pH 7.2) containing 150 mM NaCl. A total amount of 3.5 mg of purified protein was used in seven subcutaneous injections of a rabbit to produce antibodies in the ‘Animal Production and Experimentation Center’, Universidad de Sevilla (Seville, Spain). Antiserum was recovered at several times up to 5 months after the first injection and stored at -80°C until used. Antibodies specific for NblA were purified from the serum by affinity chromatography on immobilized His-tagged NblA using the AminoLink Plus^^®^^ Immobilization Kit (Thermo Fisher).

Electrophoresis mobility shift assays (EMSA) were carried out as described (Muro-Pastor et al., 1999) with a 295-bp PCR fragment amplified with oligonucleotides 153 and 183 and encompassing positions -239 to +50 with respect to the TSS of *nsrR1*. A mutated version of the same DNA fragment was obtained by overlap-extension PCR using mutagenic complementary oligonucleotides 418 and 419 and flanking oligonucleotides 153 and 183.

For Western blot analysis, *E. coli* cells were resuspended in SDS-PAGE loading buffer and the proteins fractionated on 15% SDS-PAGE. Antibodies against NblA (see above), GFP (Roche) and *E. coli* GroEL (Sigma-Aldrich) were used. The ECL Plus immunoblotting system (GE Healthcare) was used to detect the different antibodies using anti-rabbit (Sigma-Aldrich) or anti-mouse (Bio-Rad) horse-radish peroxidase conjugated secondary antibodies.

### Reporter Assays for *in vivo* Verification of Targets

For the experimental target verification in *E. coli*, we used the reporter system described (Urban and Vogel, 2007) and the superfolder GFP (sfGFP) plasmid pXG10-SF (Corcoran et al., 2012). The 5′-UTR of *nblA*, from the TSS at position -106 with respect to the initiation codon (coordinates 5407397) to 60 nucleotides within the coding region, containing the predicted NsrR1 interaction sequence, was amplified from genomic DNA using primers 253 and 254. The information about the TSS was taken from Mitschke et al. (2011). The corresponding PCR product was cloned into the vector pXG-10-SF using NsiI and NheI, resulting in a translational fusion of a truncated NblA protein with sfGFP (plasmid pIAE11, **Supplementary Table S4**). For NsrR1 expression in *E. coli*, the *nsrR1* gene was amplified from genomic DNA using primers 197 (5′-phosphorylated) and 198. The PCR product was digested with XbaI and fused to a plasmid backbone that was amplified from pZE12-luc with primers PLlacOB and PLlacOD and digested with XbaI, rendering pAVN1 (**Supplementary Table S5**).

For the mutagenesis of NsrR1 and the 5′-UTR of *nblA*, plasmids like those harboring the native versions were constructed using, in addition to the above-mentioned oligonucleotides, overlapping PCR with primers containing the desired changes (**Supplementary Tables S2, S4, S5**). Positions for mutations were selected on the basis of lowered hybridization energies predicted by IntaRNA (Mann et al., 2017). For testing various combinations of both plasmids, these were introduced into *E. coli* DH5α. Plasmid pJV300 was used as a control expressing an unrelated RNA. Fluorescence measurements were done by flow cytometry or with a microplate reader (Varioskan) using liquid cultures from eight individual colonies of each combination of plasmids, as described previously (Wright et al., 2013).

### *In vitro* Synthesis and Labeling of RNA

RNA transcripts were generated *in vitro* with MEGAscript High Yield Transcription Kit (AM1333, Ambion). The DNA templates for the transcription of NsrR1 (wild type or Mut-63 variant) and *nblA* 5′-UTR RNA (wild type and variant Comp-63) were generated by PCR with a forward primer that includes a T7 promoter sequence and three extra Gs upstream the 5′-end of the coded RNA, and a reverse primer matching the 3′ end of the RNA (**Supplementary Tables S2, S6**). The *nblA* 5′-UTR fragment extends from the TSS at position -106 to 60 nucleotides downstream the translational start. After transcription, RNAs were treated with DNase I and purified by phenol and chloroform extraction, ethanol-precipitated at -20°C, and washed with 70% ethanol.

For end labeling, 100 pmol of wild type or mutated variants of NsrR1 RNA was dephosphorylated with one unit of rAPid Alkaline Phosphatase (Roche) at 37°C for 1 h in a 20 μl reaction, followed by purification and precipitation as above. 20 pmol of dephosphorylated RNA was 5′-labeled in a 15 μl reaction with 2 μl of [γ-^32^P] ATP (10 mCi/ml, 3,000 Ci/mmol) and 15 units of polynucleotide kinase (Thermo) for 1 h at 37°C. Unincorporated nucleotides were removed with G-25 spin columns and the labeled RNAs were purified on a denaturing 8% polyacrylamide gel. The labeled RNA was visualized with a Cyclone Storage Phosphor System and the RNA bands excised and eluted overnight at 4°C in 300 μl of 20 mM Tris-HCl (pH 7.5), 0.25 M sodium acetate, 0.25% SDS, 1 mM EDTA. The eluate was ethanol-precipitated at -20°C, washed with 70% ethanol and resuspended in water.

### *In vitro* Structure Probing and Footprinting

0.1 pmol of labeled NsrR1 RNA was mixed in 7 μl with different amounts of unlabelled *nblA* 5′-UTR RNA, denatured for 1 min at 95°C and chilled on ice for 5 min, followed by addition of 1 μl of 1 mg/ml yeast RNA (Ambion AM7118) and 1 μl of 10x structure buffer (Ambion). The samples were incubated further for 15 min at 37°C.

For RNase treatment, 1 μl of 0.01 U/ml RNAse T1 (Ambion AM2283) or 1 μl of 0.01 U/ml RNAse A was added and the samples incubated for 15 min at room temperature. Reactions were stopped by addition of 20 μl of inactivation/precipitation buffer (Ambion) and incubation at -20°C for 15 min. The precipitate was washed with 70% ethanol and resuspended in 3–7 μl of denaturing formamide loading buffer.

Lead acetate treatment was performed by the addition of 1 μl of 25 mM lead(II) acetate (Sigma) and incubation for 1 min at 37°C. Reactions were stopped by addition of 1 μl 0.1 M EDTA and 22 μl of inactivation/precipitation buffer (Ambion) and incubation at -20°C for 15 min. The precipitate was washed with 70% ethanol and resuspended in 3–7 μl of denaturing formamide loading buffer.

Alkaline ladder was obtained by incubating 0.2 pmol of 5′-labeled RNA at 95°C for 3 min in 7.5 μl of alkaline hydrolysis buffer (Ambion) containing 1.5 μg of yeast RNA (Ambion AM7118). Reactions were stopped by the addition of 15 μl of denaturing formamide loading buffer.

RNase T1 G ladders were obtained by incubating 0.1 pmol of 5′-labeled RNA and 1 μl of 1 mg/ml yeast RNA (Ambion AM7118) in 9 μl sequencing buffer (Ambion) for 10 min at 50°C, followed by the addition of 1 μl of 0.1 U/ml RNAse T1 (Ambion AM2283) and incubation at room temperature for 15 min. Reactions were stopped by the addition of 20 μl of inactivation/precipitation buffer (Ambion) and incubation at -20°C for 15 min. The precipitate was washed with 70% ethanol and resuspended in 3–7 μl of denaturing formamide loading buffer.

All samples were run on 10% polyacrylamide, 7 M urea gels and bands visualized with a Cyclone Storage Phosphor System.

## Results

### Identification of NsrR1 (Nitrogen Stress-Regulated RNA 1) in *Nostoc* sp. PCC 7120

A nitrogen stress-responsive TSS was identified by differential RNA-Seq at position 2050703f of the *Nostoc* sp. PCC 7120 chromosome, in the 710-bp intergenic region between two annotated open reading frames (*alr1709* and *all1710*). Transcription from this TSS is significantly repressed in the absence of combined nitrogen (log_2_ fold-change = -2.1) (Mitschke et al., 2011). Another RNA-Seq experiment available for *Nostoc* sp. PCC 7120 also showed nitrogen-regulated transcription in the intergenic region between *alr1709* and *all1710* (Flaherty et al., 2011).

**FIGURE 1:**
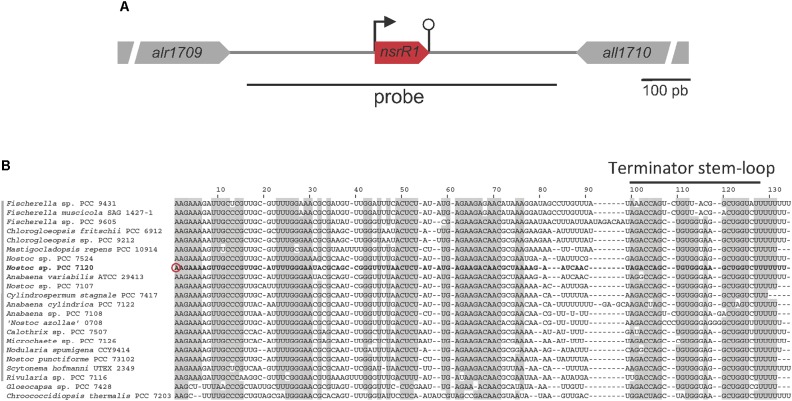
The nitrogen-stress repressed RNA 1 (NsrR1) in cyanobacteria. **(A)** Schematic representation of the region encoding NsrR1 in *Nostoc* sp. PCC 7120. The two flanking annotated open reading frames are indicated together with the region used as a probe in Northern blots (black line under the scheme). The bent arrow represents the transcriptional start at position 2050703f, and the stem-loop represents the transcriptional terminator. **(B)** Alignment of NsrR1 homologs identified in Brenes-Álvarez et al. (2016). The sequence from *Nostoc* sp. PCC 7120 is shown in bold. The verified TSS in *Nostoc* sp. PCC 7120 (Mitschke et al., 2011) is marked with a red circle and the terminator stem-loops with a black bar. Heterocystous strains are indicated with a gray bar on the left. Shadowed nucleotides indicate more than 90% conservation of the sequence.

Furthermore, a recent approach to the global identification of phylogenetically conserved small non-coding RNAs in *Nostoc* sp. PCC 7120 (Brenes-Álvarez et al., 2016) identified a putative conserved small RNA transcribed from the TSS at position 2050703f to a predicted Rho-independent terminator that ends at position 2050814f (Ionescu et al., 2010). We named the predicted sRNA NsrR1 (nitrogen stress-repressed RNA 1), not to be confused with the previously described NsiR1 (Ionescu et al., 2010). A schematic representation of the *nsrR1* genomic region in *Nostoc* sp. PCC 7120 is shown in **Figure 1A**. Sequences putatively encoding homologs of NsrR1 were found in the genomes of heterocystous strains and two unicellular strains that are phylogenetically close to the heterocystous strains, but not in more distantly related cyanobacteria (Brenes-Álvarez et al., 2016). The alignment of the corresponding sequences allows the identification of putative Rho-independent transcriptional terminators in all cases, suggesting transcription of a phylogenetically conserved small RNA (**Figure 1B**).

To determine whether a small non-coding RNA was in fact transcribed in this region, Northern blot hybridization was performed using a strand specific DNA probe that covered the intergenic region between *alr1709* and *all1710* shown in **Figure 1A**.

**FIGURE 2:**
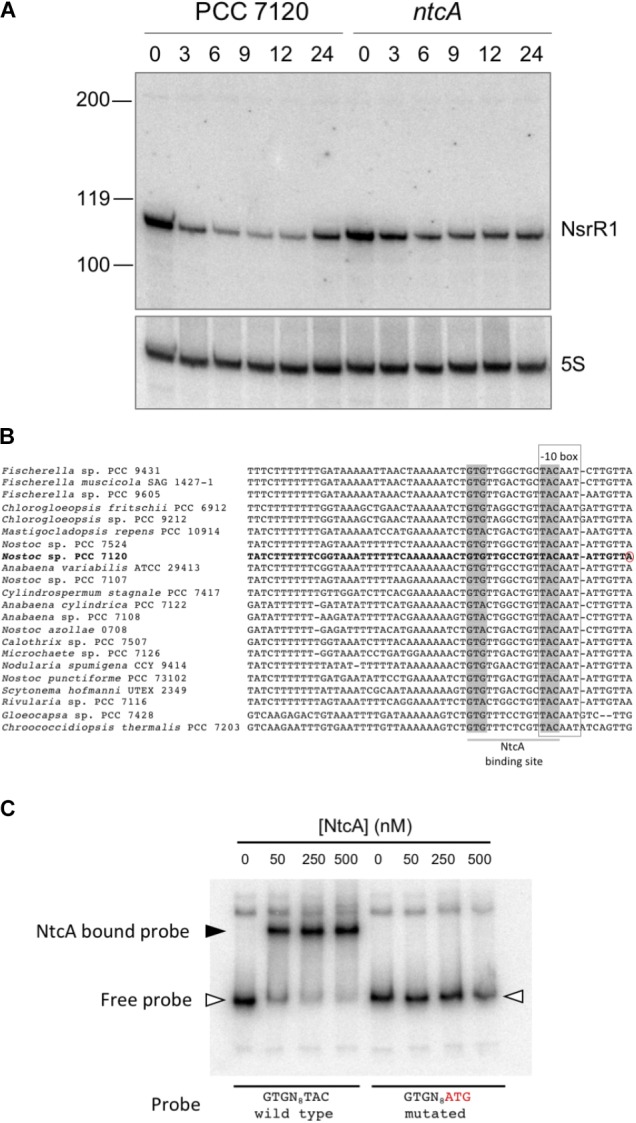
Transcription of NsrR1 is regulated by NtcA. **(A)** Nitrogen-responsive expression of NsrR1 in *Nostoc* sp. PCC 7120 and its *ntcA* mutant derivative CSE2. Expression was analyzed in cells grown in the presence of ammonium and transferred to medium containing no source of combined nitrogen (N_2_) for the number of hours indicated. The upper panel shows hybridization to the NsrR1 probe. The lower panel shows hybridization to a probe for 5S RNA used as loading and transfer control. Size markers are indicated on the left. **(B)** Comparison of promoter sequences upstream of *nsrR1* homologs encoded in cyanobacterial genomes. The verified TSS in *Nostoc* sp. PCC 7120 (Mitschke et al., 2011) is marked with a red circle. Putative NtcA binding sites (highlighted in gray) and –10 boxes (framed) are indicated. **(C)** Electrophoretic mobility shift assays showing binding of purified His-tagged NtcA protein to a DNA fragment containing the wild type promoter of NsrR1 from *Nostoc* sp. PCC 7120 or a mutated version altered in the positions indicated in red. The position of retarded DNA fragments is indicated with a black triangle, whereas the free fragments are indicated with empty triangles.

**Figure 2A** shows expression of a small RNA of about 110 nt, consistent with RNA-Seq data and the prediction for transcriptional terminators. Upon removal of combined nitrogen, transcription was strongly repressed (90% after 9 h without nitrogen, **Supplementary Figure S1**). In the *ntcA* mutant strain repression was significantly lower at all time points (**Figure 2A** and **Supplementary Figure S1**), suggesting transcriptional repression operated by NtcA. The observation that repression, although to a lower extent, took place in the *ntcA* mutant strain suggests that NtcA is not the only component repressing *nsrR1*. Transcription of NsrR1 seemed de-repressed in the wild-type after 24 h of nitrogen stress, a situation in which heterocysts have already differentiated and atmospheric nitrogen is being fixed so that nitrogen stress is alleviated. We have also analyzed the time-course of NsrR1 transcription upon ammonium addition to cells that were stably growing with nitrate or dinitrogen as nitrogen sources. In both cases addition of ammonium led to de-repression of NsrR1 expression (**Supplementary Figure S2**). De-repression was faster upon ammonium addition to cells growing in the presence of nitrate (**Supplementary Figure S2A**) than upon ammonium addition to cells growing without a source of combined nitrogen (**Supplementary Figure S2B**).

### NtcA Binds to the Promoter of NsrR1

Cyanobacterial nitrogen-responsive promoters are in many cases directly regulated by binding of the global nitrogen regulator NtcA. We have compared the promoter of NsrR1 from *Nostoc* sp. PCC 7120 with the sequences upstream from the homologs shown in **Figure 1B**. In all cases a putative NtcA binding motif (GTRN_8_TAC) partially overlaps the -10 box (**Figure 2B**), suggesting NtcA might directly repress transcription of NsrR1 by binding to these sequences. EMSA assays were performed with purified histidine-tagged NtcA and a DNA fragment extending from -239 to +50 with respect to the TSS of NsrR1 that included the putative NtcA binding sequence. A retarded band was observed that was not detected when the NtcA binding sequence was mutated (TAC changed to ATG) (**Figure 2C**). These results demonstrate sequence-specific binding of NtcA to sequences overlapping the -10 box of the promoter region of NsrR1. These observations are compatible with direct repression of transcription from the NsrR1 promoter by NtcA, although some other mechanism must be responsible for the additional repression that is still observed in the *ntcA* mutant upon nitrogen depletion (**Figure 2A**). **Supplementary Figure S3** shows a comparison of the position of the NtcA binding site in the promoter of *nsrR1* and in three previously described NtcA-repressed promoters.

### NsrR1 Interacts With *nblA* mRNA 5′-UTR

**FIGURE 3:**
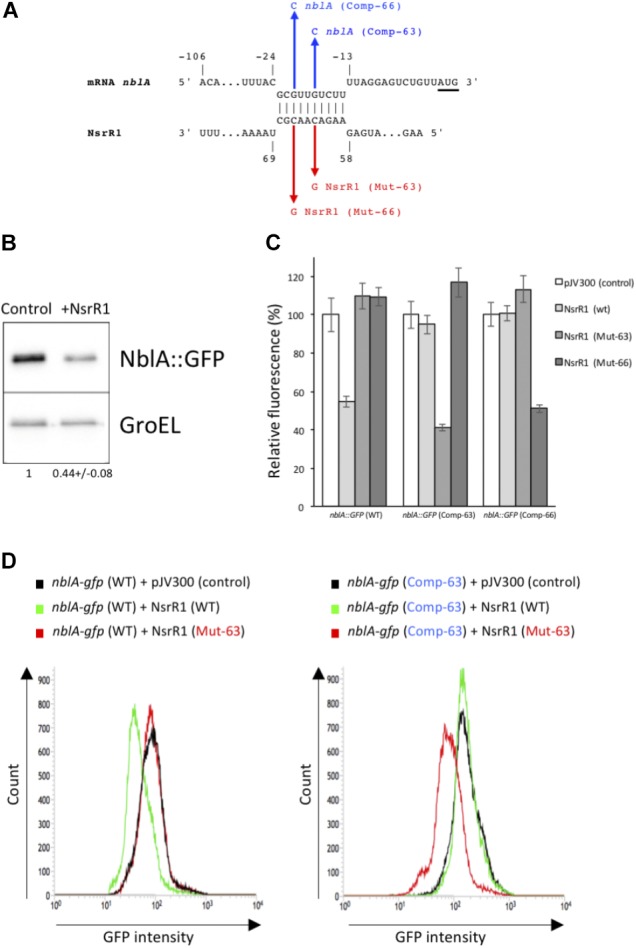
Verification of NsrR1 interaction with the 5′-UTR of *nblA* using an *in vivo* reporter system. **(A)** Predicted interaction between NsrR1 and the 5′-UTR of *nblA* according to CopraRNA (Wright et al., 2013, 2014). *nblA* nucleotides are numbered with respect to the start of the coding sequence (AUG codon is underlined). Mutations introduced in NsrR1 at position 63 (C to G, Mut-63) or 66 (C to G, Mut-66) and the corresponding compensatory mutations in *nblA* 5′-UTR position –18 (G to C, Comp-63) or –21 (G to C, Comp-66) are indicated in red and blue, respectively. **(B)** Accumulation of GFP protein in *E. coli* DH5α cultures bearing an *nblA*::sf*gfp* fusion combined with plasmid pJV300 (encoding a control RNA) or a plasmid encoding NsrR1. Western blots were carried out using antibodies against GFP or GroEL. Numbers at the bottom of the image indicate relative GFP levels with respect to control after normalization with GroEL (average of two experiments). **(C)** Fluorescence measurements of *E. coli* DH5α cultures with various combinations of plasmids expressing different versions of NsrR1 or *nblA*::sf*gfp* fusions. Plasmid pJV300 (encoding a control RNA) was used as control. The data are presented as the mean ± standard deviation of cultures from eight independent colonies after subtraction of fluorescence in cells bearing pXG-0. **(D)** FACS-based reporter assays. *E. coli* DH5α cells carrying combinations of plasmids bearing wild-type and mutated version Mut-63 of NsrR1 and wild type or mutated version Comp-63 of *nblA*::sf*gfp* were grown to stationary phase and subjected to flow cytometry analysis. Data were acquired on 50,000 cells per sample.

To identify possible mRNAs regulated by NsrR1, we have used the CopraRNA algorithm, that considers folding, energy of hybridization and phylogenetic conservation of the sRNA (Wright et al., 2013, 2014) (**Table 1**). Among the 20 best targets predicted by CopraRNA, *nblA* is the gene with a known function that has the highest score (*p*-value = 8.45 × 10^-7^). NsrR1 is predicted to interact with the 5′-UTR of *nblA* mRNA (**Figure 3A**). A potential interaction between NsrR1 and the 5′-UTR of *nblA* mRNA is conserved in many cyanobacteria that contain NsrR1 (**Supplementary Figure S4**). Although the exact position in the 5′-UTR predicted to interact with NsrR1 is not conserved, in most cases it is very close or overlapping the putative Shine-Dalgarno sequence.

**Table 1:** CopraRNA prediction of candidate mRNAs that interact with NsrR1.

fdr	*p*-value	Locus tag	Annotation
2.63 × 10^-05^	6.11 × 10^-09^	*all1871*	Unknown
**1**.**58** ×**10**^-^**^03^**	**8.45 × 10^-07^**	***asr4517***	***nblA***
1.58 × 10^-03^	1.10 × 10^-06^	*all1043*	Hypothetical protein
7.46 × 10^-02^	9.11 × 10^-05^	*all7229*	Unknown
7.46 × 10^-02^	1.04 × 10^-04^	*asr2666*	Hypothetical protein
9.92 × 10^-02^	1.61 × 10^-04^	*alr3099*	GCN5-related *N*-acetyltransferase
9.97 × 10^-02^	1.85 × 10^-04^	*all4613*	Acetohydroxy acid synthase; *IlvG*
0.110	2.63 × 10^-04^	*alr4291*	Photosystem II CP43 protein
0.110	2.92 × 10^-04^	*alr5103*	LL-Diaminopimelate aminotransferase apoenzyme
0.110	3.04 × 10^-04^	*alr2079*	Cl- channel, voltage gated
0.110	3.05 × 10^-04^	*alr3594*	Response regulator receiver domain protein (*CheY*)
0.136	4.40 × 10^-04^	*alr4814*	Hypothetical protein
0.136	4.51 × 10^-04^	*all3970*	Endonuclease III
0.136	4.75 × 10^-04^	*all3794*	Tryptophan synthase beta subunit
0.141	5.23 × 10^-04^	*all0330*	GAF sensor signal transduction histidine kinase
0.162	6.40 × 10^-04^	*all7229*	Unknown
0.165	6.92 × 10^-04^	*all1228*	Hypothetical protein
0.165	7.26 × 10^-04^	*alr0309*	Hypothetical protein
0.305	1.43 × 10^-03^	*alr4953*	Hypothetical protein
0.305	1.49 × 10^-03^	*alr4696*	Class I peptide chain release factor

*CopraRNA (Wright et al., 2013, 2014) was implemented at the Freiburg RNA Tools Server with default options. NsrR1 from *Nostoc* sp. PCC 7120 was used as reference together with the NsrR1 sequences from *Anabaena cylindrica* PCC 7122, *Anabaena variabilis* ATCC 29413 (recently renamed *Trichormus variabilis* NIES-23), *Cylindrospermum stagnale* PCC 7417, *Nodularia spumigena* CCY9414, ‘*Nostoc azollae*’ 0708, *Nostoc punctiforme* PCC 73102, *Nostoc* sp. PCC 7524, *Rivularia* sp. PCC 7116, *Nostoc* sp. PCC 7107, and *Calothrix* sp. PCC 7507. The *nblA* gene studied in this work is shown in bold. Only the 20 genes with the highest scores are shown*.

To verify the interaction between NsrR1 and the mRNA of *nblA*, we used a heterologous reporter assay (Corcoran et al., 2012), in which the shortest 5′-UTR of *nblA*, corresponding to transcription from position -106 (Mitschke et al., 2011) plus 60 nucleotides of the coding sequence of *nblA* were fused to the gene for sfGFP and co-expressed with NsrR1 or with a control RNA in *E. coli*. According to CopraRNA, the predicted interacting region (**Figure 3A**) was located very close to the translation initiation region of *nblA* and therefore was predicted to affect translation of the mRNA. Accumulation of GFP protein was measured in cells bearing different combinations of plasmids. Cells carrying the *nblA*::sf*gfp* translational fusion showed significant GFP fluorescence, as well as GFP protein determined by Western blot (**Figure 3B**), demonstrating that the translation initiation region of *nblA* was functional in *E. coli*. The GFP fluorescence of cells bearing the *nblA::*sf*gfp* fusion (and the amount of GFP protein) decreased to about 50% of the control when NsrR1 was co-expressed, indicating a direct interaction between NsrR1 and the 5′-UTR of *nblA*, which affects translation (**Figures 3B,C**). To verify the interaction at the predicted site, two different point mutations were introduced in NsrR1 in the middle of the predicted helix between NsrR1 and the 5′-UTR of *nblA* (nucleotide 63, C to G; nucleotide 66, C to G). Compensatory mutations were introduced in the corresponding positions of the 5′-UTR of *nblA* (nucleotide 89, G to C; nucleotide 86, G to C). Mutation of either one of those nucleotides in NsrR1 drastically reduced the interaction between NsrR1 and the 5′-UTR of *nblA*, as suggested by the absence of fluorescence reduction with respect to control (**Figures 3C,D**). When the mutated version of the sRNA was combined with the mutated version of the mRNA containing the compensatory change, base pairing was restored and again a significant fluorescence reduction was observed. These data support a direct interaction of NsrR1 with the 5′-UTR of the *nblA* mRNA and its effect on translation of NblA.

**FIGURE 4:**
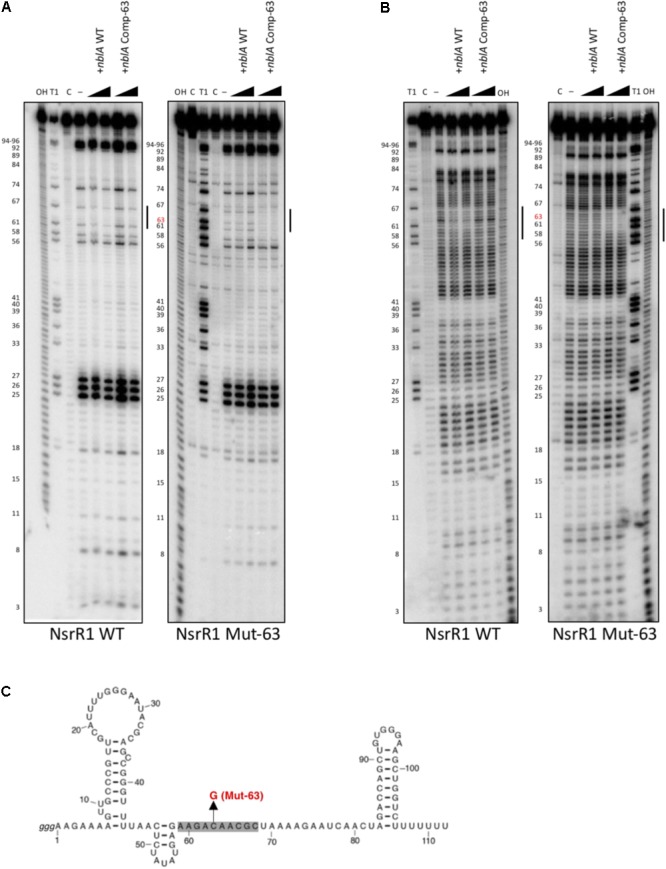
*In vitro* footprinting assay of the NsrR1/*nblA* 5′-UTR interaction. **(A)** RNase T1 footprinting of the interaction between wild type NsrR1 (left) or mutant C63G (Mut-63, right) with wild type and compensatory mutant (Comp-63) of the *nblA* 5′-UTR. **(B)** Pb(II) footprinting of the interaction between wild type NsrR1 (left) or mutant C63G (right) with wild type and compensatory mutant (Comp-63) of the *nblA* 5′-UTR. Two concentrations of *nblA* RNA were used (100 and 200 nM). The protected area is indicated by vertical bars in **(A,B)**. NsrR1 was 5′ end-labeled. C, untreated control; OH, alkaline ladder; T1, RNase T1 ladder. Nucleotide positions of NsrR1 are shown on the left. **(C)** Secondary structure model of NsrR1 from *Nostoc* sp. PCC 7120 based on structure probing (**Supplementary Figure S5**). The area protected by *nblA* RNA is shaded. The nucleotide changed in Mut-63 is also indicated.

In addition, we have studied the interaction between NsrR1 and the *nblA* mRNA by *in vitro* footprinting analysis. ^32^P-labeled NsrR1 was incubated with unlabeled *nblA* mRNA (a fragment extending from positions -106 to +60 with respect to the start of the coding sequence) and probed with RNase T1 (**Figure 4A**) or lead(II) acetate (**Figure 4B**). When wild type NsrR1 RNA and *nblA* mRNA were used, a footprint was detected between positions 58 and 67 of NsrR1, in agreement with the bioinformatic prediction (**Figure 3A**). This footprint was not observed with the mutant version of *nblA* mRNA (Comp-63). However, when the mutant version of NsrR1 RNA (Mut-63) was used, only the mutant *nblA* mRNA (Comp-63) generated a footprint, but not the wild type *nblA* mRNA. These *in vitro* results are therefore in agreement with the *in vivo* results obtained in *E. coli* with the GFP fusion analysis.

We have also performed *in vitro* structure probing analysis of NsrR1 (**Supplementary Figure S5**). The NsrR1 region involved in interaction with *nblA* mRNA is predicted to be single stranded, according to the pattern of sensitivity to RNase T1, RNase A, and lead(II) acetate (**Figure 4** and **Supplementary Figure S5**).

### *nblA* Expression Is Reduced by NsrR1 in *Nostoc* sp. PCC 7120

Transcription of NsrR1 is only partially (and transiently) repressed upon nitrogen step down. Therefore, to study *in vivo* in *Nostoc* the possible effect of NsrR1 on *nblA* expression, we have generated a mutant strain lacking NsrR1 (Δ*nsrR1*) by deleting the DNA region containing *nsrR1* through homologous recombination. Cells lacking NsrR1 have no apparent phenotype under normal growth conditions. Furthermore, there is no growth difference with respect to wild-type cells in medium containing nitrate, ammonia, or lacking a nitrogen source (**Supplementary Figure S6**). A complemented strain (Δ*nsrR1 +* P*_petE_*::*nsrR1*) in which NsrR1 expression is under control of the Cu^2+^-inducible *petE* promoter (Buikema and Haselkorn, 2001) was generated, releasing NsrR1 expression from nitrogen control and allowing Cu^2+^-controlled expression instead.

**FIGURE 5:**
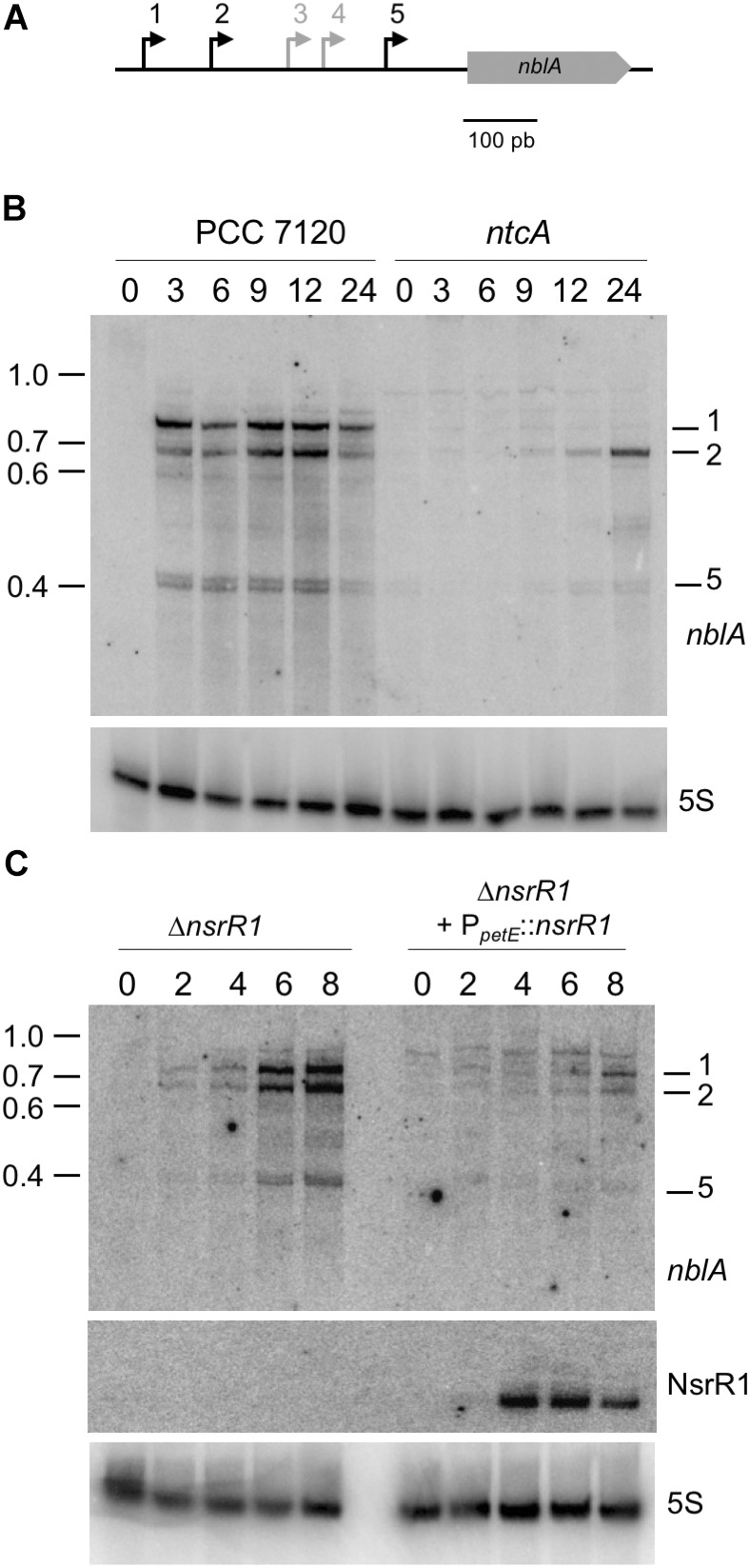
Nitrogen-regulated transcripts of *nblA* and their regulation by NsrR1 in *Nostoc* sp. PCC 7120. **(A)** Schematic representation of the five transcriptional start sites (bent arrows) previously defined for *nblA* (Mitschke et al., 2011), with the nitrogen-regulated transcriptional starts (1, 2, and 5) indicated in black. **(B)** Identification by Northern hybridization of the transcripts originated from nitrogen-regulated transcriptional starts in *Nostoc* sp. PCC 7120 and its *ntcA* mutant derivative CSE2. Expression was analyzed in cells grown in the presence of ammonium and transferred to medium containing no source of combined nitrogen for the number of hours indicated. The upper panel shows hybridization to the *nblA* probe. The lower panel shows hybridization to a probe for 5S RNA used as loading and transfer control. Size markers (kb) are indicated on the left. Transcripts corresponding to TSS 1, 2, and 5 are indicated. **(C)** Nitrogen-regulated expression of *nblA* in strains with altered levels of NsrR1. Expression was analyzed in a mutant strain lacking NsrR1 (Δ*nsrR1*) and in the same strain complemented with a plasmid bearing *nsrR1* under control of the Cu^2+^-regulated promoter of the *petE* gene. Cells were grown in the presence of ammonium in medium lacking Cu^2+^ (to prevent expression of the *petE* promoter) and transferred to medium containing no source of combined nitrogen and 1.5 μM Cu^2+^ for the number of hours indicated. The upper panel shows hybridization to the *nblA* probe. The middle panel shows hybridization to the probe for NsrR1 indicated in **Figure 1A**. The lower panel shows hybridization to a probe for 5S RNA used as loading and transfer control.

*nblA* is transcribed from five TSS, three of them induced in response to nitrogen deprivation (Mitschke et al., 2011) (see a scheme in **Figure 5A**). Northern blot hybridization confirmed the NtcA dependent induction of transcription from three of the *nblA* promoters (**Figure 5B**). We have compared *nblA* expression between the Δ*nsrR1* and the Δ*nsrR1 +* P*_petE_*::*nsrR1* strains during several hours after nitrogen step down (so that transcription of *nblA* is induced) and Cu^2+^ addition (to induce transcription of NsrR1 from the *petE* promoter in strain Δ*nsrR1 +* P*_petE_*::*nsrR1*) (**Figure 5C**). Under these conditions transcription of all three N-regulated *nblA* transcripts was induced in the Δ*nsrR1* strain in response to nitrogen deficiency. However, accumulation of the three transcripts was strongly reduced in the strain that produced NsrR1 from the *petE* promoter (**Figure 5C**). After 8 h in the absence on combined nitrogen the amount of *nblA* mRNA (adding all three transcripts together) in the Δ*nsrR1* strain is about fourfold higher than in the Δ*nsrR1 +* P*_petE_*::*nsrR1* strain (**Supplementary Figure S7**), indicating that NsrR1 has a significant negative effect on the accumulation of *nblA* mRNA in *Nostoc*.

To analyze the accumulation of NblA protein in the different strains, we have generated antibodies against recombinant purified *Nostoc* NblA protein. Unfortunately, although these antibodies could detect NblA in Western blots of extracts from *E. coli* cells overproducing NblA (**Supplementary Figure S8**), they were, however, unable to detect NblA in extracts of *Nostoc* under conditions of full induction of *nblA* expression. Similarly, a histidine-tagged version of NblA could not be detected in *Nostoc* using commercial anti-His antibodies. Lack of detection of NblA with either antibodies could likely be due to the presence of very low levels of NblA in cyanobacterial cells. In fact, it has been suggested that NblA is itself degraded by the cellular degradation machinery (Baier et al., 2014; Sendersky et al., 2014).

**FIGURE 6:**
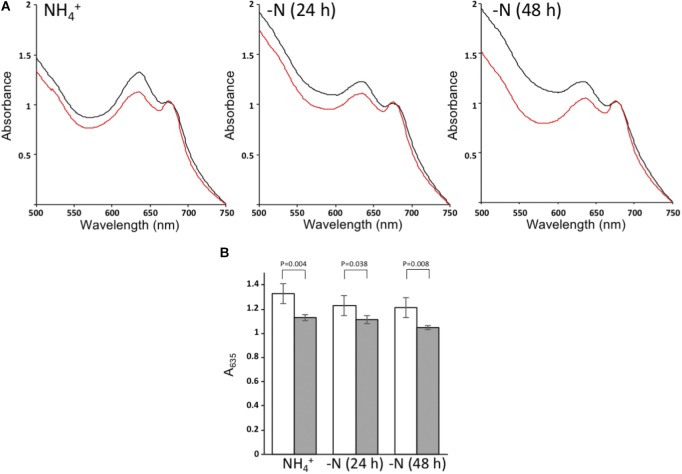
Whole cell absorption spectra. **(A)** Absorption spectra of wild type cells (black) or Δ*nsrR1* cells (red) growing in the presence of NH_4_^+^ (left) or after 24 h (center) or 48 h (right) of nitrogen depletion, normalized by the chlorophyll content (A_680_). **(B)** Normalized A_635_ of cultures from wild type cells (white) or Δ*nsrR1* cells (gray) growing in the presence of NH_4_^+^, or after 24 h or 48 h of nitrogen depletion. Error bars represent the standard error of four experiments. *p*-values (unpaired *t*-test) are indicated for the comparison of wild type and Δ*nsrR1* cells at each time point.

To assess possible effects of the Δ*nsrR1* mutation on pigment content, whole cell spectra were taken of cells growing in the presence of NH_4_^+^ or 24 and 48 h after removal of combined nitrogen. Δ*nsrR1* mutant cells have lower phycocyanin content in all cases, even in the presence of NH_4_^+^ (**Figure 6**). Phycocyanin content was further reduced in both strains by nitrogen starvation but while in the wild type strain it is similar at 24 and 48 h, in the Δ*nsrR1* mutant strain the reduction is stronger at 48 h than at 24 h (**Figure 6B**).

## Discussion

Small regulatory RNAs are important players in the operation of regulatory effects exerted by classical transcription factors. In *E. coli*, every major transcription factor regulates transcription of one or several regulatory RNAs that, in turn, operate post-transcriptional regulation. FNR and CRP, the best studied members of the CRP/FNR family, are known to regulate the expression of several small regulatory RNAs, including CRP-induced CyaR (Papenfort et al., 2008; De Lay and Gottesman, 2009), CRP-repressed Spot 42 (Beisel and Storz, 2011) or FNR-induced FnrS (Durand and Storz, 2010), all of them contributing to the regulatory effects exerted by these two transcriptional regulators.

In response to nitrogen deficiency, expression of many cyanobacterial genes is regulated, either positively or negatively, by the global nitrogen regulator NtcA, a CRP-family regulator. In many cases the mechanism underlying such regulation seems to be direct activation or repression of gene expression, as deduced from the presence of consensus NtcA binding sites in the respective promoters. However, in cases where no obvious sequence elements can be identified as responsible for the observed regulation, the mechanism is assumed to be indirect. Transcriptomic analyses identifying NtcA-regulated non-coding RNAs suggest this type of molecules might also be involved in NtcA-mediated post-transcriptional regulation (Mitschke et al., 2011; Brenes-Álvarez et al., 2016). A feed-forward coherent regulatory loop involving NtcA and a nitrogen-regulated sRNA has been recently described in *Synechocystis* sp. PCC 6803. In this unicellular strain NtcA operates transcriptional regulation of the genes encoding glutamine synthetase and its inactivating factors IF7 and IF17, and also regulates transcription of NsiR4 (nitrogen stress inducible RNA 4), that modulates translation of IF7 (Klähn et al., 2015). A similar loop has been described for RpaB, that operates direct transcriptional regulation of several genes encoding proteins of the photosystem I and of PsrR1 (photosynthesis regulatory RNA 1), a high light-responsive small RNA that post-transcriptionally regulates translation of some of those photosystem I-related proteins (Georg et al., 2014). These regulatory loops, like the case of PmgR1 (photomixotrophic growth RNA 1), a sRNA that acts downstream of PmgA and is involved in regulation of glycogen accumulation (de Porcellinis et al., 2016), point to a relevant role of small regulatory RNAs in the regulation of cyanobacterial metabolism.

Here, we describe NsrR1, a small RNA whose transcription is regulated in response to nitrogen limitation in *Nostoc* sp. PCC 7120. Repression of NsrR1 transcription is at least partially operated by NtcA, as deduced from Northern analysis in the *ntcA* mutant and binding assays with purified NtcA protein (**Figure 2**). The observation that repression by NtcA is only partial suggests participation of some other regulator(s) integrating different signal(s). In this context, expression of NsrR1 might be additionally regulated by another repressor, perhaps in the CRP family. Conservation of a T immediately upstream of the GTN_10_AC conserved NtcA binding sequence suggests possible binding of an unidentified CRP-like regulator (Forcada-Nadal et al., 2014). In contrast to the wide distribution observed for some cyanobacterial sRNAs such as Yfr1 (Voss et al., 2007; Brenes-Álvarez et al., 2016), NsrR1 homologs are only found in the genomes from heterocystous cyanobacteria (and closely related unicellular strains), suggesting its possible function might be specifically related to some particular metabolic trait in this group of organisms.

Phycobilisomes account for a large amount of the total protein, and therefore nitrogen, in cyanobacterial cells. In unicellular cyanobacteria unable to fix N_2_, recycling of phycobiliproteins as a source of amino acids is an adaptive response to lack of available combined nitrogen, prior to cessation of growth. In heterocystous cyanobacteria, that are ultimately able to overcome nitrogen deficiency by nitrogen fixation, transcription of *nblA* is induced as a transient response to nitrogen deprivation but the extension of PBS degradation is much lower than in unicellular strains (Maldener et al., 1991; Baier et al., 2004). Using an *in vivo* reporter system established in *E. coli* we demonstrate a direct interaction between NsrR1 and the 5′-UTR of *nblA*, leading to reduced expression of an NblA::sfGFP fusion in the presence of NsrR1. Unfortunately, we have not succeeded in detecting the NblA protein in extracts from *Nostoc* sp. PCC 7120, and therefore cannot assess directly the effects of NsrR1 overexpression on NblA accumulation at the protein level. Antibodies generated and purified against NblA from *Nostoc* sp. PCC 7120 detected NblA when expressed in *E. coli* (**Supplementary Figure S8**) but not in cyanobacterial extracts, probably because of limited amount of NblA in these cells.

We have also demonstrated reduced accumulation of *nblA* transcripts in strains of *Nostoc* sp. PCC 7120 that over-express NsrR1 (**Figure 5** and **Supplementary Figure S7**), which can be a consequence of the interaction of these two RNA molecules leading to reduced translation of NblA. Interaction of NsrR1 at the predicted region in the 5′-UTR of *nblA* has been verified by *in vitro* footprinting experiments (**Figure 4**). The predicted region targeted is located close to the translation initiation and is therefore common to all five possible *nblA* 5′-UTRs. Thus, although transcriptional regulation of *nblA* might be operated by different regulators integrating different signals, all possible transcripts would be susceptible to additional post-transcriptional regulation by NsrR1 and therefore the accumulation of NblA protein would be ultimately under control of nitrogen limitation. The reduced accumulation of *nblA* mRNAs in the presence of NsrR1 can be attributed to destabilization of the mRNA when translation is inhibited. A more direct mechanistic effect of NsrR1 on mRNA stability, recruitment of a ribonuclease for instance, cannot be excluded.

The presence of NsrR1 in ammonium growing cells would suppress transcriptional noise due to leaky transcription initiation of *nblA*, reducing background production of NblA that would have undesirable consequences on phycobilisome stability. In fact, RNA-Seq and primer extension assays (Mitschke et al., 2011) indicate above-background transcription of *nblA* from promoters 2, 4, and 5 in ammonium growing cells, that is not readily detectable in the less sensitive Northern blot experiments (**Figure 5**). Another protein directly involved in PBS degradation, NblB, has been shown to be present under nitrogen sufficient conditions in *Synechococcus elongatus* (Levi et al., 2018). The RNA-Seq data available for *Nostoc* sp. PCC 7120 (Flaherty et al., 2011; Mitschke et al., 2011) suggest that in this organism *nblB* is also expressed constitutively. Therefore, it would be of utmost importance to avoid any background accumulation of NblA under nitrogen sufficient conditions that, together with NblB, would compromise PBS stability. In addition, the presence of NsrR1 would lead to a delay in NblA production after NtcA induction of *nblA* transcription due to a threshold effect (Levine and Hwa, 2008), creating a delay in the onset of phycobilisome degradation and providing stability by avoiding an inappropriate response under fluctuating environmental conditions. Controlled degradation of PBS, although not essential for adaptation to transient nitrogen deficiency in N_2_-fixing strains, might be an advantage in natural environments subjected to changes in different factors such as light intensity or availability of other nutrients.

**FIGURE 7:**
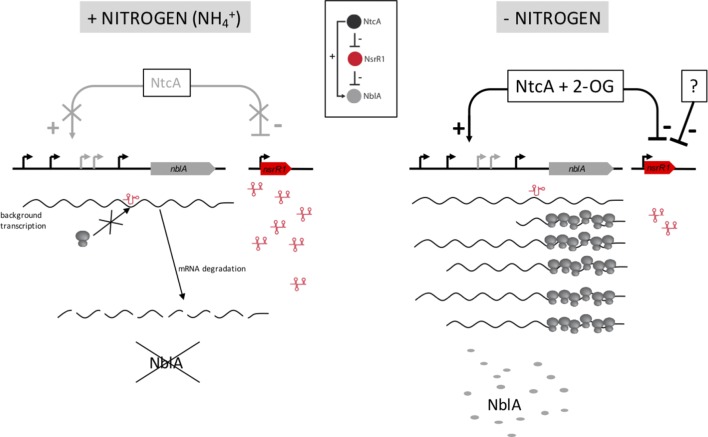
Model for the function of NsrR1 in *nblA* regulation. Under nitrogen sufficient conditions **(Left)** intracellular 2-oxoglutarate (2-OG) concentration is low and NtcA cannot activate transcription of *nblA*, while NsrR1 is expressed at high levels. Therefore, under these conditions NsrR1 is in excess of any small amount of *nblA* mRNA that could be present due to background transcription. Binding of NsrR1 to the mRNA blocks translation, resulting in degradation of the mRNA and preventing any production of NblA protein under nitrogen sufficient conditions. When the nitrogen source is removed **(Right)** 2-OG concentration increases and NtcA can activate transcription of *nblA*, while *nsrR1* is partially repressed. As a result, there is not enough NsrR1 RNA to repress translation of an excess of *nblA* transcripts and NblA protein can accumulate. Inset: Scheme of a type 4 feed-forward loop (Nitzan et al., 2017) as applied to the NtcA-NsrR1-NblA regulatory circuit.

The changes detected in pigment content between wild type and Δ*nsrR1* mutant cells agree with the proposed repression of NblA accumulation by NsrR1, and with our model of regulation (**Figure 7**). We speculate that the slightly, but significant, lower phycobilin/chlorophyll ratio of the Δ*nsrR1* mutant compared with wild type (**Figure 6B**) when growing in the presence of NH_4_^+^ is due to background expression of *nblA* in the absence of NsrR1, resulting in reduced phycobilin stability. After 48 h of nitrogen depletion heterocysts are fully functional and nitrogen status should be more similar to NH_4_^+^ growing cells. In fact, in wild type cells the phycocyanin/chlorophyll ratio is stabilized while in the Δ*nsrR1* it drops further, suggesting extended presence of NblA in the absence of NsrR1.

The regulatory circuit we propose (**Figure 7**) represents a type 4 coherent feed-forward loop (Alon, 2007; Nitzan et al., 2017). This type of loop, in which a transcription factor is the top regulator, provides a delayed increase in the level of the target protein (NblA in this case) when the transcription factor (NtcA in this case) is activated, and an accelerated decrease in target protein level when the transcription factor is deactivated (Beisel and Storz, 2011). Dual regulation at the transcriptional and post-transcriptional levels should provide strict control of the levels of the NblA proteolytic adaptor. In addition, because the top regulator (NtcA) is itself subjected to differential accumulation in heterocysts (Olmedo-Verd et al., 2006; Sandh et al., 2014), the feed-forward loop is likely reinforced in heterocysts *vs.* vegetative cells. Higher amounts of NtcA in heterocysts could promote stronger transcription of *nblA*. The combination of increased transcription of *nblA* with low levels of NsrR1 (affecting its translation) would lead to increased amounts of NblA protein producing faster PBS degradation specifically in developing heterocysts.

## Author Contributions

IÁ-E contributed to the acquisition, analysis, or interpretation of the data. AM-P and AV contributed to the acquisition, analysis, interpretation of the data, and writing of the manuscript.

## Conflict of Interest Statement

The authors declare that the research was conducted in the absence of any commercial or financial relationships that could be construed as a potential conflict of interest.
