# Supplementary material for: NsrR1, a Nitrogen Stress-Repressed sRNA, Contributes to the Regulation of nblA in Nostoc sp. PCC 7120
Source: Front Microbiol. 2018 Sep 24;9:2267. doi: 10.3389/fmicb.2018.02267 (PMC6166021; doi:10.3389/fmicb.2018.02267)
Supplement: Supplementary file 1 [file Data_Sheet_1.PDF]

NsrR1, a Nitrogen Stress-Repressed sRNA, Contributes to the Regulation of *nblA* in *Nostoc* sp. PCC 7120

Isidro Álvarez-Escribano, Agustín Vioque*, Alicia M. Muro-Pastor

Instituto de Bioquímica Vegetal y Fotosíntesis, Consejo Superior de Investigaciones Científicas and Universidad de Sevilla, Sevilla, Spain

***Correspondence:**
Dr. Agustín Vioque, Instituto de Bioquímica Vegetal y Fotosíntesis, Consejo Superior de Investigaciones Científicas and Universidad de Sevilla, Avda. Américo Vespucio 49, Sevilla, 41092, Spain
vioque@us.es

Keywords:
Regulatory RNA, cyanobacteria, NtcA, feed-forward loop, post-transcriptional regulation

Running title: sRNA regulation of *nblA*

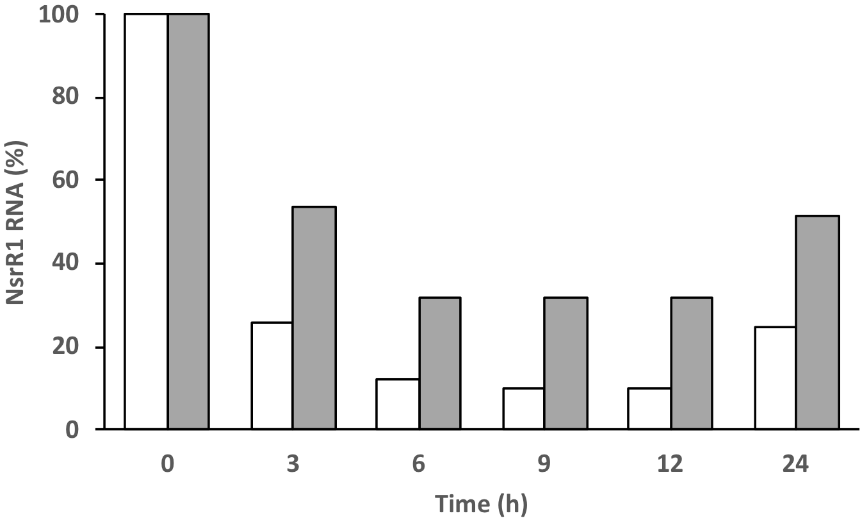

Figure S1. Quantification of NsrR1 expression. The signal corresponding to NsrR1 in the Northern blot shown in Fig. 2A for the wild type strain (white) or the *ntcA* strain (grey) were quantified and normalized to the amount of 5S RNA in each lane. NsrR1 amount is expressed as percentage of the amount present at time 0 in each strain.

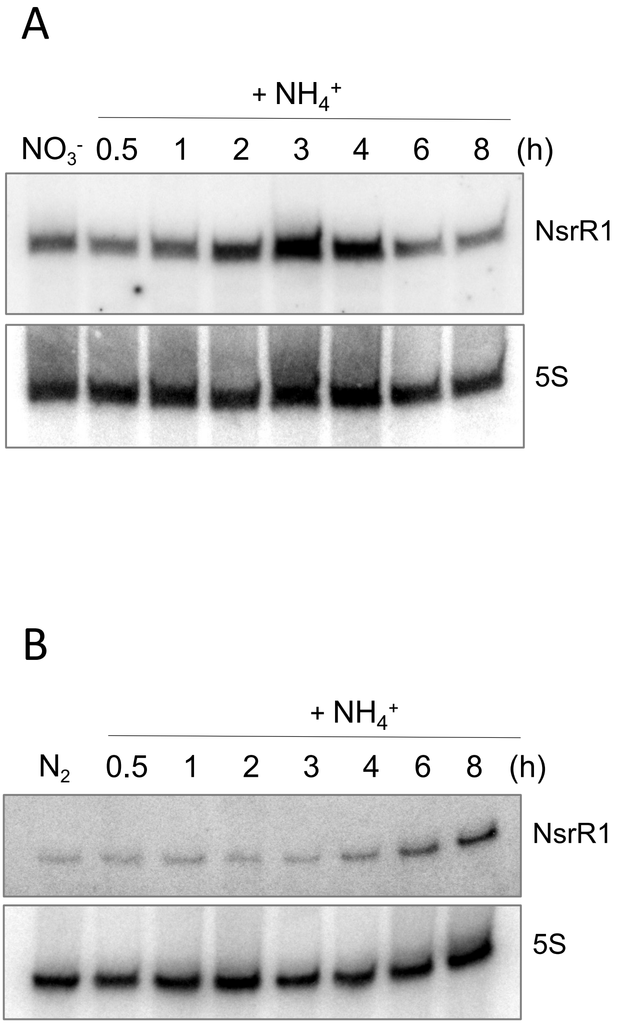

Figure S2. Expression of NsrR1 upon addition of ammonium. Expression was analyzed in cultures of *Nostoc* sp. PCC 7120 growing at the expense of nitrate **(A)** or N_2 **(B)** to which 10 mM NH_4^+ was added. RNAs were extracted at the indicated times (h) after addition of NH_4^+. Upper panels show hybridization to the NsrR1 probe. Lower panels show hybridization to a probe for 5S RNA used as loading and transfer control.

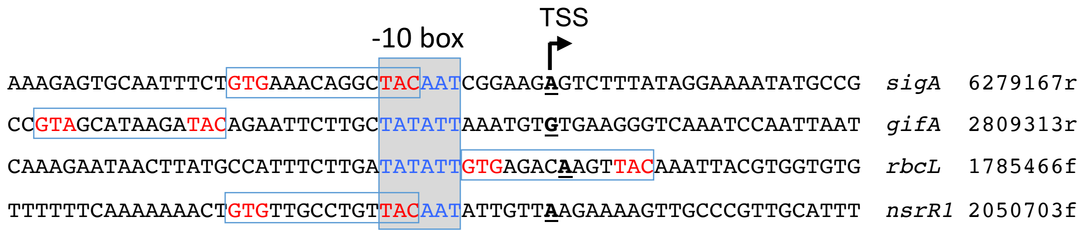

Figure S3. NtcA binding sites on NtcA repressed promoters. The promoter of *nsrR1* is shown together with the NtcA-repressed promoters of *sigA* (Muro-Pastor et al., 2017), *gifA* (Galmozzi et al., 2010) and *rbcL* (Ramasubramanian et al., 1994). Promoters were aligned by the -10 promoter element (grey box). The TSS (Mitschke et al., 2011) are highlighted in bold and underlined. The NtcA binding sites are framed.

***Fischerella* sp. PCC 9605**

```
            -157                                    -120
nblA mRNA    |                                       |
5'-UGG...UUAGU    AA   U CA        G              G  AAACA...UUU-3'
              CAAA  AUC C    CCAAAG GU AUGGGCAAUUU UC
              ||||  ||| |    |||||: |: |:||||||||| ||
              GUUU  UAG G    GGUUUU CG UGCCCGUUAAA AG
3'-UUU...UUUGG    A    U CAAG      G  U           A  AA-5'
NsrR1        |                                       |
             41                                      2
```

***Chlorogloeopsis fritschii* PCC 6912**

```
             43                        64
nblA mRNA    |                         |
5'-GGU...CAGCA   G               A     GUACA...UCU-3'
              UUC UUCUUU CG    CU CUCAA
              ||| |||||: ||    || |||||
              AAG AAGAAG GC    GA GAGUU
3'-UUU...UUUUA          C  AACA  A     AUCUC...GAA-5'
NsrR1        |                         |
             77                        52
```

***Chlorogloeopsis* sp. PCC 9212**

```
             43                        64
nblA mRNA    |                         |
5'-GGU...CAGCA   G               A     GUACA...UCU-3'
              UUC UUCUUU CG    CU CUCAA
              ||| |||||: ||    || |||||
              AAG AAGAAG GC    GA GAGUU
3'-UUU...UUUUA          C  AACA  A     AUCUC...AAU-5'
NsrR1        |                         |
             78                        53
```

***Mastigocladopsis repens* PCC 10914**

```
            -172                    -148
nblA mRNA    |                       |
5'-UGG...AAAUU   U U       AA        CCAUU...UUU-3'
              AGA G GUCAAGC  AAAAUUAC
              ||| | |||||:|  ||||||||
              UCU C CAGUUUG  UUUUAAUG
3'-UUU...AGAGU     U       GG        CGCAA...GAA-5'
NsrR1        |                       |
             55                      32
```

***Nostoc* sp. PCC 7524**

```
            -27                     -5
nblA mRNA    |                       |
5'-UGG...CCUUA       C               UAAUUAUG...UCU-3'
              CGCGUUG CUUUUUA  GGAGUC
              ||||||| |||:|:|  :|||||
              GCGCAAC GAAGAGU  UCUCAG
3'-UUU...AGUAA       A       UA      UUUUG...AAU-5'
NsrR1        |                       |
             70                      46
```

***Nostoc* sp. PCC 7107**

```
            -38                         -10
nblA mRNA    |                           |
5'-UAU...CCCGC     CUC      A            GGAGUUUACUAUG...UUU-3'
              UAAAU   UUUUUU CGCGUUGUUUUC
              :||||   |||||| ||||||||:|||
              GUUUA   AAAAAA GCGCAACAGAAG
3'-UUU...AGAUA     C                      AGUUA...AAU-5'
NsrR1        |                           |
             83                          58
```

***Cylindrospermum stagnale* PCC 7417**

```
            -26                                                    26
nblA mRNA    |                                                     |
5'-GGG...CUUAA              C          CUAUGAGCC          AA U     UAGAA...UUU-3'
              CGCGUUGUUUUUUU   GGAGUUAA         AACC AAUUG  C UUCCC
              ||||||||:||:|:   :|||||||         |||| |||||  | |||||
              GCGCAACAGAAGAG   UCUCAAUU         UUGG UUAAC  G AAGGG
3'-UUU...AAAAA              UUA                     U     GC C     UUUUA...AAU-5'
NsrR1        |                                                     |
             70                                                    25
```

***Anabaena cylindrica* PCC 7122**

```
            -25         -13
nblA mRNA    |           |
5'-CCA...UUUUA  A        UUUGGUUUUAAUUAUG...UUU-3'
              CG GUUGUUUU
              || |||||:||
              GC CAACAGAA
3'-UUU...AACAA  G        GAGUU...AAU-5'
NsrR1        |           |
             70          58
```

***Anabaena* sp. PCC 7108**

```
            -24       -14
nblA mRNA    |         |
5'-CUU...UUUUA         UUUUGGUUUUAACUAUG...UUU-3'
              CGCGUUGUU
              ||||||||:
              GCGCAACAG
3'-UUU...AACAA         AAGAG...AAU-5'
NsrR1        |         |
             70        60
```

***Nostoc azollae* 0708**

```
            -24                                                 26
nblA mRNA    |                                                  |
5'-AAU...UAUAA                UUUAACUAU  A              A  U     UAGAA...UUU-3'
              GUGUUGUUUUCUCGGU         GA UCA  ACC AAUUG GC UUCCU
              |||||||:|||||::|         || |||  ||| ||||| || ||||:
              CACAACAGAAGAGUUA         CU AGU  UGG UUAAC CG AAGGG
3'-UGG...ACAAG                U          C   UU   U     G  C     UUUUA...GAA-5'
NsrR1        |                                                  |
             68                                                 24
```

***Calothrix* sp. PCC 7507**

```
            -27                                 9
nblA mRNA    |                                    |
5'-UUG...CAUUA              UC         AUUAU      ACCAA...UUU-3'
              CGCGUUGUUUUCUU    GGAGUUA     GAGCCA
              ||||||||:||||:    :||||||     :|||||
              GCGCAACAGAAGAG    UCUCAAU     UUCGGU
3'-UUU...AAAAA              UUAA                  UUAAC...GAA-5'
NsrR1        |                                    |
             70                                   38
```

***Microchaete* sp. PCC 7126**

```
            -26                                 10
nblA mRNA    |                                    |
5'-UUG...UUUAA             UC         AUUAU       CCAAU...UUU-3'
              GCGUUGUUUUCUU    GGAGUUA     GAGUCAA
              |||||||:||||:    :||||||     |||:|||
              CGCAACAGAAGAG    UCUCAAU     CUCGGUU
3'-UUU...AAAAG             UUAA                   UAACG...GAA-5'
NsrR1        |                                    |
             69                                   37
```

***Nodularia spumigena* CCY 9414**

```
            -26                     -4
nblA mRNA    |                        |
5'-UAG...UUUUA        U               UAUUAUG...UUU-3'
              CC CGUUG UUUUCUC  GGAGUU
              || ||||| |:|||||  :|||||
              GG GCAAC AGAAGAG  UCUCAA
3'-UUU...AAAAA  C             UA      UUUUA...GAA-5'
NsrR1        |                        |
             68                       44
```

***Nostoc punctiforme* PCC 73102**

```
            -24                       -2
nblA mRNA    |                         |
5'-UUG...CUUUA                         CUAUG...UUU-3'
              UGCGUU UUUUUUC   GGAGUUAA
              |||||| |:||:||   :|||||||
              ACGCAA AGAAGAG   UCUCAAUU
3'-UUU...AUAAA      C       UUC        UUGGG...AAU-5'
NsrR1        |                         |
             70                        44
```

***Scytonema hofmanni* UTEX 2349**

```
            -22                        1
nblA mRNA    |                         |
5'-UGG...UUAUU                         AUGAG...UUU-3'
              CGUUGUCUUUU    GGAGUUAAUU
              |||||||||:|    :||||||||:
              GCAACAGAAGA    UCUCAAUUAG
3'-UUU...AAAUU           GUUU          GCUUA...AAG-5'
NsrR1        |                         |
             67                        41
```

***Chroococcidiopsis thermalis* PCC 7203**

```
            -31                  -17
nblA mRNA    |                    |
5'-AUA...CAACU                    UGUUUUCAGGAGUUAUAUG...UCC-3'
              CAGUC AC      UACGUU
              ||||| ||      ||||||
              GUCAG UG      AUGCAA
3'-UUU...AUCAG     U  AAUAUA      CAGCC...GAA-5'
NsrR1        |                    |
             85                   64
```

Figure S4. Conservation of the predicted interaction between the *nblA* mRNA and NsrR1 in cyanobacteria. Potential interaction between NsrR1 and *nblA* mRNA (analyzed from 200 nucleotides upstream of the start codon to the stop codon of the NblA coding sequence) was computed for each cyanobacteria shown in Fig. 1 using IntaRNA software (Mann et al., 2017). Nucleotide positions in the *nblA* mRNA are numbered from first nucleotide of the coding sequence, negative upstream to positive downstream. AUG start codons (red) and putative Shine-Dalgarno sequences (green shading) are indicated. Only those strains with a predicted stable interaction are shown.

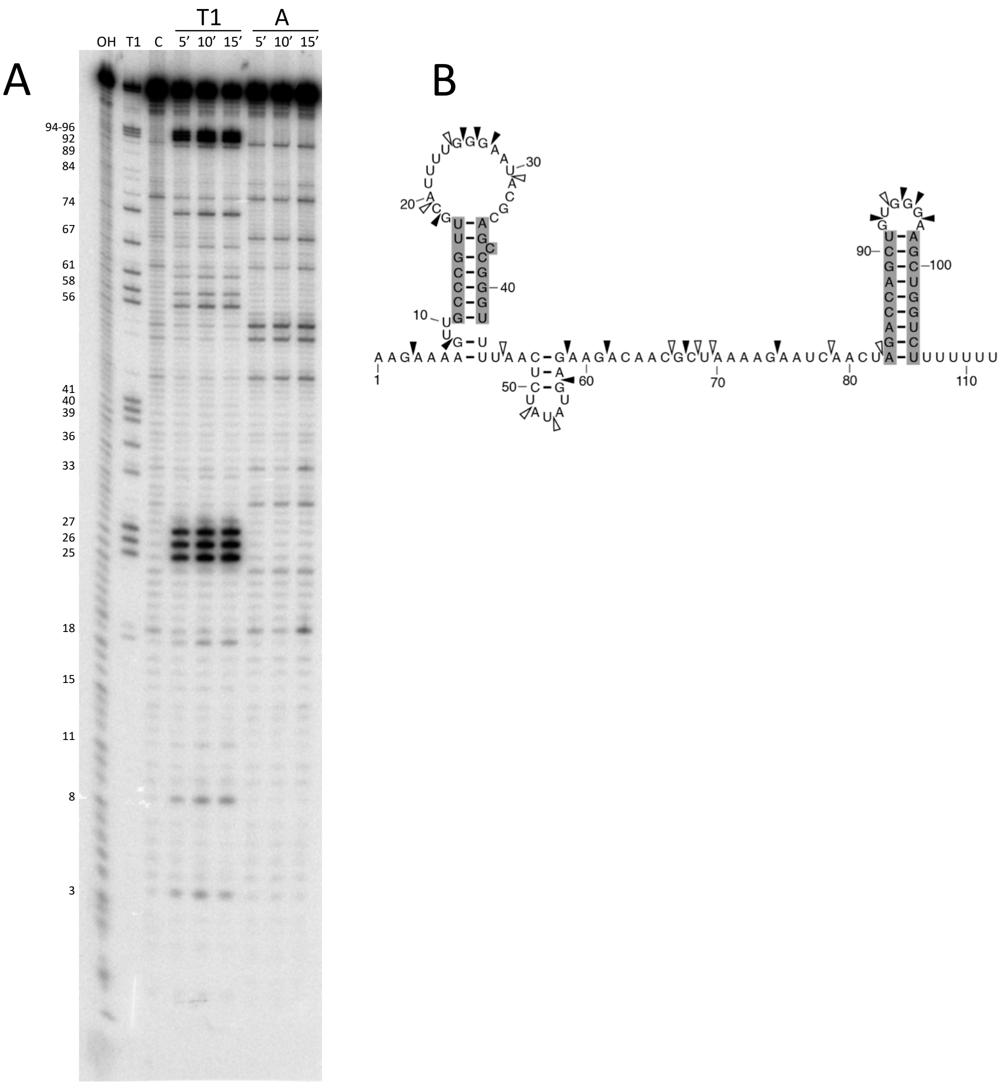

Figure S5. Structure probing of NsrR1. (A) 5'-end labelled NsrR1 was incubated with RNase T1 (1 mU/ml) or RNase A (1 mU/ml) for 5, 10 or 15 minutes and the resulting fragments analyzed on a 8% polyacrilamide sequencing gel. C, untreated control; OH, alkaline ladder; T1, RNase T1 ladder. Nucleotide positions of NsrR1 are shown on the left. **(B)** Secondary structure model of NsrR1. Black and white triangles indicate the positions sensitive to hydrolysis by RNase T1 and RNase A, respectively. The shaded areas highlight the nucleotides more resistant to lead(II)-induced hydrolysis (Figure 4).

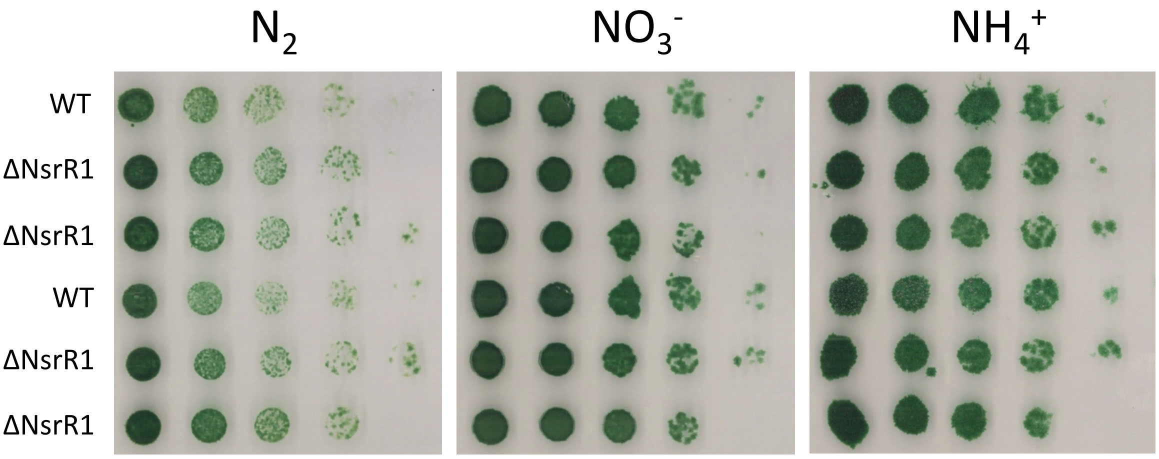

Figure S6. Growth properties of Δ*nsrR1* strains. Cells were grown in the presence of nitrate, resuspended in $BG11_0$ at an OD_{750}= 0.3. Five-fold serial dilutions of liquid cultures of wild type or 4 different Δ*nsrR1* isolates were prepared and 10 µl of each dilution plated on $BG11_0$ plates lacking nitrogen (N_2), or containing nitrate (NO_3^-) or ammonium (NH_4^+). Pictures were taken after 10 days of incubation at 30ºC.

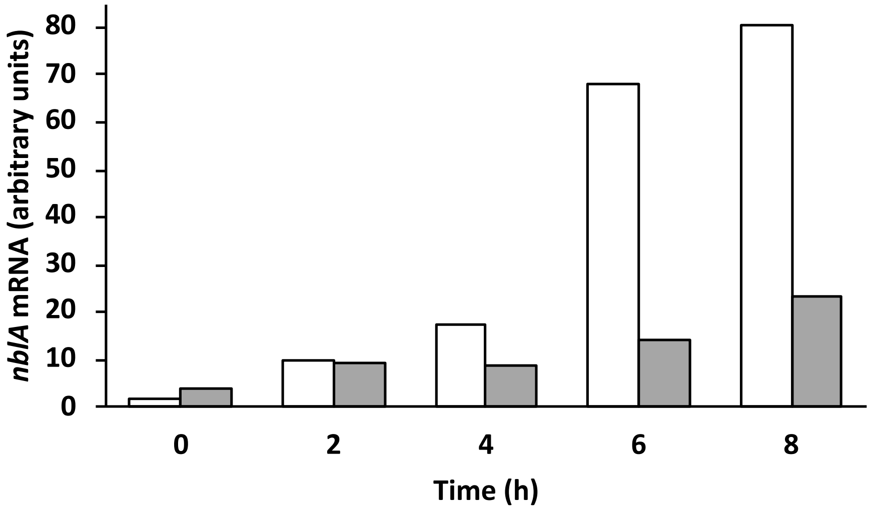

Figure S7. Quantification of *nblA* mRNA expression. The signals corresponding to bands 1, 2, and 5 in the Northern blots shown in Fig. 5C for the Δ*nsrR1* strain (white) or the Δ*nsrR1*+ P_{petE}::*nsrR1* strain (grey) were quantified and their total amount normalized to the amount of 5S RNA in each lane. Similar qualitative results were obtained in two other experiments.

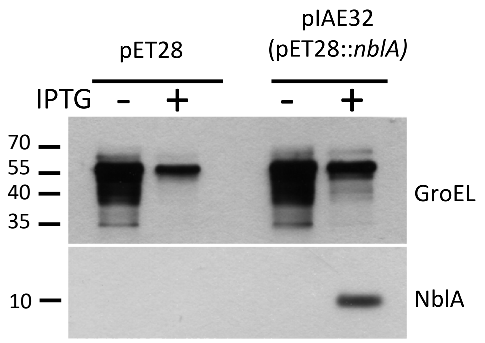

Figure S8. Detection of NblA in *E. coli* cells using antibodies against NblA. *E. coli* cells containing vector pET28 or a pET28-derived construct expressing the *nblA* gene were used for Western detection with antibodies generated against purified NblA. Antibodies against GroEL were used as control. Position of protein size markers (kDa) is shown on the left.

Table S1. Strains

	Description	Reference
Escherichia coli		
DH5α	Used for routine transformation	(Hanahan, 1983)
BL21(DE3)-RIL	Cm^R, used for overexpression of recombinant proteins	Agilent Technologies
Nostoc sp.		
PCC 7120	Wild type	Pasteur Culture Collection
CSE2	*ntcA* null mutant	(Frías et al., 1994)
Δ*nsrR1*	*nsrR1* gene deleted	This work
Δ*nsrR1*+P_{petE}::*nsrR1*	*nsrR1* gene under control of the *petE* promoter in Δ*nsrR1* background	This work

Table S2. Oligonucleotides

Name	Sequence (5'-3')	Used for
153	GAGTTAAAACCCGGCTGCGTATTCCC	PCR of NsrR1 promoter region (-239 to +50)
183	ATCGATACTCTCGCAACAACG	PCR of NsrR1 promoter region (-239 to +50)
418	CCTGTATGAATATTGTTAAGAAAAGTTGCC	PCR to generate a mutated NtcA binding site in the NsrR1 promoter
419	CAATATTCATACAGGCAACACAG	
158	CATCTGCCTCTGCCTCTTCTG	PCR of NsrR1 to generate radioactive probe
159	CCTTCCTTGTAGGCAGTCGAG	
170	GGATCCAACAGACCAACGCTGAG	Deletion of NsrR1
171	TAAAACGACTCGAGGTAATCAGCCTCTATC	
172	TGATTACCTCGAGTCGTTTTACGTGGTGTC	
173	GGATCCTAGTCTCACACCATTAGG	
184	CTCGAGACACCACGTAAAACGAC	Cloning of NsrR1 under the promoter of *petE*
185	TAAAATGAAATAAAGAAAAGTTGCCCGTTGC	
186	GCAACTTTTCTTTATTTCATTTTAAATAAAATCGACACC	
299	GTTTTATCGATGGACTCAGAACACAGTACTC	
189 (PLlacOB)	CGCACTGACCGAATTCATTAA	Plasmid backbone amplification from pZE12-luc
190 (PLlacOD)	GTGCTCAGTATCTTGTTATCCG	
197	5'P-AAGAAAAGTTGCCCGTTGC	PCR of NsrR1 for cloning in PZE12-luc
198	GTTTTTTCTAGACAGAGACACCACGTAAAACGACT	
253	GTTTTATGCATACAGAGGAATAATCAACAATATGGG	PCR of 5'-UTR of *nblA* for cloning in pXG10-SF
254	GTTTTGCTAGCGGTGGCAAATGAGCGAATGC	
315	GAAGAGAACGCTAAAAGAATCAACTAGACC	Mutagenesis of NsrR1 to generate Mut-63
316	GCGTTCTCTTCTCATATAGAGTTAAAACCC	
317	GACAAGGCTAAAAGAATCAACTAGACCAGC	Mutagenesis of NsrR1 to generate Mut-66
318	CTTTTAGCCTTGTCTTCTCATATAGAGTTAAAACCC	
329	GCGTTCTCTTTTAGGAGTCTGTTATGAACC	Mutagenesis of *nblA* to generate Comp-63
330	CCTAAAAGAGAACGCGTAAAGGTTATGTCG	
331	CCTTTACGCCTTGTCTTTTAGGAGTCTG	Mutagenesis of *nblA* to generate Comp-66
332	GACAAGGCGTAAAGGTTATGTCGG	
343	GTTTTCCATGGTCGTGACACAAGATAAGGCC	PCR of *ntcA* for cloning in pET28a
344	GTTTTCTCGAGAGTGAACTGTCTGCTGAGAG	
358	GTTTTCATATGAACCAACCAATCGAATTGTCATTAG	PCR of *nblA* for cloning in pET28a
359	GTTTTCTCGAGCTATGCCGGAGTGGAACCAGAATC	
569	GTTTTGAATTC**TAATACGACTCACTATA**GGGAAGAAAAGTTGCCCGTTGC	PCR of template for *in vitro* transcription of NsrR1
570	GTTTTGGATCCTTTAAAAAAAGACCAGCTTCCCAC	
571	GTTTTGAATTC**TAATACGACTCACTATA**GGGACAGAGGAATAATCAACAATATGG	PCR of template for *in vitro* transcription of *nblA* 5'-UTR
572	GTTTTGGATCCGGTGGCAAATGAGCGAATG	

Sequences are given in 5'→3' direction; 5'P denotes a 5' monophosphate. Underlined, restriction sites used for cloning. In red nucleotide changes with respect to the native wild type sequence. The T7 promoter sequence in oligonucleotides 569 and 571 is in bold.

Table S3. Plasmids

Name	Description	Reference
pET28a(+)	Km^R, Vector for his-tagged protein expression	Novagen
pSpark	Ap^R, Vector for cloning PCR products	Canvax Biotech
pCSRO	Sm^RSp^R, *sacB*-containing vector for conjugation of *Nostoc*	(Merino-Puerto et al., 2010)
pXG0	Cm^R, control plasmid without GFP	(Urban and Vogel, 2007)
pXG10-SF	Cm^R, vector for the generation of sfGFP fusions	(Corcoran et al., 2012)
pZE12-luc	Ap^R, plasmid for cloning sRNAs	(Lutz and Bujard, 1997)
pAVN1	Ap^R, PCR fragment generated with primers 197 and 198, containing *nsrR1*, digested with XbaI and cloned in the vector backbone generated by PCR of pZE12-luc with primers 189 (PLlacOB) and 190 (PLlacOD)	This work
pIAE11	Cm^R, PCR fragment generated with primers 253 and 254, containing *nblA* 5'-UTR+60 bp, digested with NsiI and NheI, and cloned in pXG10-SF digested with the same enzymes	This work
pIAE17	ClaI-XhoI fragment from pSAM329 cloned into pSAM221	This work
pIAE26	Ap^R, same as pAVN1 but with a C to G change at position 63 of NsrR1 (mut-63)	This work
pIAE27	Ap^R, same as pAVN1 but with a C to G change at position 66 of NsrR1 (mut-66)	This work
pIAE28	Cm^R, same as pIAE11 but with a G to C change at position 89 of the 5'-UTR (Comp-63)	This work
pIAE29	Cm^R, same as pIAE11 but with a G to C change at position 86 of the 5'-UTR (Comp-66)	This work
pIAE32	Km^R, PCR fragment generated with primers 358 and 359, containing the *nblA* gene from *Nostoc*, digested with NdeI and XhoI and cloned in pET28a(+) digested with the same enzymes	This work
pSAM221	Sm^RSp^R, derivative of pSAM200 (Ionescu et al., 2010), a replicative vector for *Nostoc*. Contains a polylinker with unique ClaI and XhoI sites inserted at the unique EcoRI site of pSAM200.	This work
pSAM319	Ap^R, pSpark derivative containing a fragment of the NsrR1 locus with the NsrR1 gene deleted	This work
pSAM325	Sm^RSp^R, pCSRO derivative containing a BamHI fragment from pSAM319 corresponding to the NsrR1 region with the NsrR1 gene deleted	This work
pSAM329	Ap^R, pSpark derivative with NsrR1 under control of the *petE* promoter	This work
pSAM334	Km^R, PCR fragment generated with primers 343 and 344, containing the *ntcA* gene from *Nostoc* digested with NcoI and XhoI and cloned in pET28a(+) digested with the same enzymes	This work

Table S4. Sequences of inserts in the *nblA-sfgfp* fusion plasmids.

Plasmid	Sequence	Description
pIAE11	atgcATACAGAGGAATAATCAACAATATGGGGCAGGTACTAAC TAAAGTCCTATGCCTGTGGGGCTTCTGTAACCGACATAACCTT TACGCGTTGTCTTTTAGGAGTCTGTTATGAACCAACCAATCGA ATTGTCATTAGAACAACAATTCAGCATTCGCTCATTTGCCACC gctagc	*nblA* WT
pIAE28	atgcATACAGAGGAATAATCAACAATATGGGGCAGGTACTAAC TAAAGTCCTATGCCTGTGGGGCTTCTGTAACCGACATAACCTT TACGCGTTCTCTTTTAGGAGTCTGTTATGAACCAACCAATCGA ATTGTCATTAGAACAACAATTCAGCATTCGCTCATTTGCCACC gctagc	*nblA* Comp-63
pIAE29	atgcATACAGAGGAATAATCAACAATATGGGGCAGGTACTAAC TAAAGTCCTATGCCTGTGGGGCTTCTGTAACCGACATAACCTT TACGCCTTGTCTTTTAGGAGTCTGTTATGAACCAACCAATCGA ATTGTCATTAGAACAACAATTCAGCATTCGCTCATTTGCCACC gctagc	*nblA* Comp-66

Nostoc gene sequences are capitalized, in which black letters correspond to 5'UTR parts and green letters to ORF parts, respectively. NsiI and NheI sites that were used for cloning are highlighted in magenta and yellow, respectively. In red nucleotide changes with respect to the native wild type sequence.

Table S5. Sequences of inserts in plasmids containing NsrR1 used for verification in *E. coli*.

Plasmid	Sequence	Description
pAVN1	AAGAAAAGTTGCCCGTTGCATTTTGGGAATACGCAGCCGGG TTTTAACTCTATATGAGAAGACAACGCTAAAAGAATCAACT AGACCAGCTGTGGGAAGCTGGTCTTTTTTTCCGTACATACA CGTTTGACCAATAGTCGTTTTACGTGGTGTCTCTGTCTAGA	NsrR1 WT
pIAE26	AAGAAAAGTTGCCCGTTGCATTTTGGGAATACGCAGCCGGG TTTTAACTCTATATGAGAAGAGAACGCTAAAAGAATCAACT AGACCAGCTGTGGGAAGCTGGTCTTTTTTTCCGTACATACA CGTTTGACCAATAGTCGTTTTACGTGGTGTCTCTGTCTAGA	NsrR1 Mut-63
pIAE27	AAGAAAAGTTGCCCGTTGCATTTTGGGAATACGCAGCCGGG TTTTAACTCTATATGAGAAGACAAGGCTAAAAGAATCAACT AGACCAGCTGTGGGAAGCTGGTCTTTTTTTCCGTACATACA CGTTTGACCAATAGTCGTTTTACGTGGTGTCTCTGTCTAGA	NsrR1 Mut-66

Grey shadowed letters indicate the *nsrR1* sequence. Modified nucleotides are marked in red. XbaI restriction site used for cloning is highlighted in blue.

Table S6. Sequences of templates used for *in vitro* transcription.

Template	Sequence
nblA Comp-63	**GGG**ACAGAGGAATAATCAACAATATGGGGCAGGTACTAACTAAAGTCCTATGCCTGTGGGG CTTCTGTAACCGACATAACCTTTACGCGTTCTCTTTTAGGAGTCTGTTATGAACCAACCAA TCGAATTGTCATTAGAACAACAATTCAGCATTCGCTCATTTGCCACCggatc
nblA WT	**GGG**ACAGAGGAATAATCAACAATATGGGGCAGGTACTAACTAAAGTCCTATGCCTGTGGGG CTTCTGTAACCGACATAACCTTTACGCGTTGTCTTTTAGGAGTCTGTTATGAACCAACCAA TCGAATTGTCATTAGAACAACAATTCAGCATTCGCTCATTTGCCACCggatc
NsrR1 Mut-63	**GGG**AAGAAAAGTTGCCCGTTGCATTTTGGGAATACGCAGCCGGGTTTTAACTCTATATGAG AAGAGAACGCTAAAAGAATCAACTAGACCAGCTGTGGGAAGCTGGTCTTTTTTT
NsrR1 WT	**GGG**AAGAAAAGTTGCCCGTTGCATTTTGGGAATACGCAGCCGGGTTTTAACTCTATATGAG AAGACAACGCTAAAAGAATCAACTAGACCAGCTGTGGGAAGCTGGTCTTTTTTT

In bold, non-encoded Gs added for efficient *in vitro* transcription. The nucleotides changed in Mut-63 and Comp-63 are in red. The initiation codon of *nblA* is underlined.

Supplementary references

Corcoran, C.P., Podkaminski, D., Papenfort, K., Urban, J.H., Hinton, J.C., and Vogel, J. (2012). Superfolder GFP reporters validate diverse new mRNA targets of the classic porin regulator, MicF RNA. *Mol. Microbiol.* 84, 428-445. doi: 10.1111/j.1365-2958.2012.08031.x.

Frías, J.E., Flores, E., and Herrero, A. (1994). Requirement of the regulatory protein NtcA for the expression of nitrogen assimilation and heterocyst development genes in the cyanobacterium *Anabaena* sp. PCC 7120. *Mol. Microbiol.* 14, 823-832. doi: 10.1111/j.1365-2958.1994.tb01318.x.

Galmozzi, C.V., Saelices, L., Florencio, F.J., and Muro-Pastor, M.I. (2010). Posttranscriptional regulation of glutamine synthetase in the filamentous cyanobacterium *Anabaena* sp. PCC 7120: differential expression between vegetative cells and heterocysts. *J. Bacteriol.* 192, 4701-4711. doi: 10.1128/JB.00222-10.

Hanahan, D. (1983). Studies on transformation of *Escherichia coli* with plasmids. *J. Mol. Biol.* 166, 557-580.

Ionescu, D., Voss, B., Oren, A., Hess, W.R., and Muro-Pastor, A.M. (2010). Heterocyst-specific transcription of NsiR1, a non-coding RNA encoded in a tandem array of direct repeats in cyanobacteria. *J. Mol. Biol.* 398, 177-188. doi: 10.1016/j.jmb.2010.03.010.

Lutz, R., and Bujard, H. (1997). Independent and tight regulation of transcriptional units in *Escherichia coli* via the LacR/O, the TetR/O and AraC/I1-I2 regulatory elements. *Nucleic Acids Res.* 25, 1203-1210. doi: 10.1093/nar/25.6.1203.

Mann, M., Wright, P.R., and Backofen, R. (2017). IntaRNA 2.0: enhanced and customizable prediction of RNA-RNA interactions. *Nucleic Acids Res.* doi: 10.1093/nar/gkx279.

Merino-Puerto, V., Mariscal, V., Mullineaux, C.W., Herrero, A., and Flores, E. (2010). Fra proteins influencing filament integrity, diazotrophy and localization of septal protein SepJ in the heterocyst-forming cyanobacterium *Anabaena* sp. *Mol. Microbiol.* 75, 1159-1170. doi: 10.1111/j.1365-2958.2009.07031.x.

Mitschke, J., Vioque, A., Haas, F., Hess, W.R., and Muro-Pastor, A.M. (2011). Dynamics of transcriptional start site selection during nitrogen stress-induced cell differentiation in *Anabaena* sp. PCC7120. *Proc. Natl. Acad. Sci. USA* 108, 20130-20135. doi: 10.1073/pnas.1112724108.

Muro-Pastor, A.M., Brenes-Alvarez, M., and Vioque, A. (2017). A combinatorial strategy of alternative promoter use during differentiation of a heterocystous cyanobacterium. *Environ. Microbiol. Rep.* 9, 449-458. doi: 10.1111/1758-2229.12555.

Ramasubramanian, T.S., Wei, T.F., and Golden, J.W. (1994). Two *Anabaena* sp. strain PCC 7120 DNA-binding factors interact with vegetative cell- and heterocyst-specific genes. *J. Bacteriol.* 176**,** 1214-1223. doi: 10.1128/jb.176.5.1214-1223.1994.

Urban, J.H., and Vogel, J. (2007). Translational control and target recognition by *Escherichia coli* small RNAs in vivo. *Nucleic Acids Res.* 35**,** 1018-1037. doi: 10.1093/nar/gkl1040.
